# Enthralling genetic regulatory mechanisms meddling insecticide resistance development in insects: role of transcriptional and post-transcriptional events

**DOI:** 10.3389/fmolb.2023.1257859

**Published:** 2023-09-06

**Authors:** Chandramohan Muthu Lakshmi Bavithra, Marimuthu Murugan, Shanmugasundaram Pavithran, Kathirvel Naveena

**Affiliations:** ^1^ Department of Agricultural Entomology, Tamil Nadu Agricultural University, Coimbatore, India; ^2^ Centre for Plant Protection Studies, Tamil Nadu Agricultural University, Coimbatore, India

**Keywords:** insects, insecticide resistance, detoxification enzymes, pathways, ncRNAs, RNA methylation

## Abstract

Insecticide resistance in insects severely threatens both human health and agriculture, making insecticides less compelling and valuable, leading to frequent pest management failures, rising input costs, lowering crop yields, and disastrous public health. Insecticide resistance results from multiple factors, mainly indiscriminate insecticide usage and mounted selection pressure on insect populations. Insects respond to insecticide stress at the cellular level by modest yet significant genetic propagations. Transcriptional, co-transcriptional, and post-transcriptional regulatory signals of cells in organisms regulate the intricate processes in gene expressions churning the genetic information in transcriptional units into proteins and non-coding transcripts. Upregulation of detoxification enzymes, notably cytochrome P450s (CYPs), glutathione S-transferases (GSTs), esterases [carboxyl choline esterase (CCE), carboxyl esterase (CarE)] and ATP Binding Cassettes (ABC) at the transcriptional level, modification of target sites, decreased penetration, or higher excretion of insecticides are the noted insect physiological responses. The transcriptional regulatory pathways such as AhR/ARNT, Nuclear receptors, CncC/Keap1, MAPK/CREB, and GPCR/cAMP/PKA were found to regulate the detoxification genes at the transcriptional level. Post-transcriptional changes of non-coding RNAs (ncRNAs) such as microRNAs (miRNA), long non-coding RNAs (lncRNA), and epitranscriptomics, including RNA methylation, are reported in resistant insects. Additionally, genetic modifications such as mutations in the target sites and copy number variations (CNV) are also influencing insecticide resistance. Therefore, these cellular intricacies may decrease insecticide sensitivity, altering the concentrations or activities of proteins involved in insecticide interactions or detoxification. The cellular episodes at the transcriptional and post-transcriptional levels pertinent to insecticide resistance responses in insects are extensively covered in this review. An overview of molecular mechanisms underlying these biological rhythms allows for developing alternative pest control methods to focus on insect vulnerabilities, employing reverse genetics approaches like RNA interference (RNAi) technology to silence particular resistance-related genes for sustained insect management.

## Introduction

Insects are the most common species on the planet, inhabiting and interacting with fauna and flora in ecological systems, including humans. Few insect species serve humans and their wellbeing by pollinating crops, scavenging garbage, and performing other tasks. On the other hand, many insect species adversely affect public health, crops, hygiene, and other sectors (Eggleton, 2020). When a biological equilibrium is disturbed, insects expand uncontrollably, wreaking havoc on humans, threatening food production, spreading human diseases, and requiring pest control treatments. The principal pest-reduction strategy employs chemicals that challenge insects and their reproduction. Insecticides are chemical or biological molecules used to kill or otherwise inhibit insects from engaging in damaging behaviors (Manyilizu, 2019).

According to the Insecticide Resistance Action Committee (IRAC) Mode of Action (MoA) Classification Version 10.5 March 2023, 36 insecticide groups are available, each of which contains a sub-group, class, or exemplifying active ingredient of the main groups (IRAC, 2023), indicating that these insecticides differ in structure, synthesis, and mode of action. The diversity of these compounds can be seen in their availability, which includes chlorinated hydrocarbons, organophosphates, carbamates, pyrethroids, neonicotinoids, formamidines, phenylpyrazoles, sulfoximines, spinosyns, juvenile hormone analogs, benzoylureas, buprofezin, cyromazine, and other molecules, as well as botanical and microbial agents (Simon, 2014; IRAC, 2023).

Long-term insecticide efficacy is crucial for successful and sustainable food and fiber production and public health. However, given numerous reports of chemical agents’ ineffectiveness in suppressing insect populations, prolonged use of insecticides has had unexpected consequences, most notably the emergence of insecticide-resistant insect pests. Insecticide resistance (IR) is common and widespread, nearly nine decades after synthetic pesticides proved popular in pest management. IR is defined as a reduction in an insect population’s susceptibility to a previously effective insecticide caused by the continued use and/or possible cross-selection with other chemical substances, which occurs through genetic, physiological, or behavioral changes and is also a hereditary trait (WHO, 2016; Oppold and Müller, 2017; IRAC, 2023).

The San Jose scale, *Comstockaspis perniciosa* Comstock (Hemiptera: Diaspididae), demonstrated the first known insecticide resistance to lime sulfur in 1914 (Melander, 1914). Since the discovery and widespread application of DDT and other synthetic insecticides in the late 1940s, the number of resistant insect species has steadily expanded. Insecticide resistance to 339 insecticides and five insecticidal characteristics expressed in genetically modified plants has been documented for 602 insect species as of 2019 (Sparks and Nauen, 2015; Sparks et al., 2019). The most notorious insect at the top of the list of resistant insects is *Plutella xylostella* (Linnaeus) (Lepidoptera: Plutellidae), which has evolved resistance to 101 different active ingredients of insecticides (APRD, 2023). Pests like the Colorado potato beetle, *Leptinotarsa decimlineata* (Say) (Coleoptera: Chrysomelidae), the two-spotted spider mite, *Tetranychus urticae* Koch (Trombidiformes: Tetranychidae), and the green peach aphid, *Myzus persicae* (Sulzer) (Hemiptera: Aphididae) are each resistant to 56, 84, and 96 different insecticides, respectively (APRD, 2023). Unsurprisingly, worldwide reports of insecticide resistance to the majority of WHO-approved public health insecticides have been made (Ranson et al., 2011; Moyes et al., 2017). Human disease vectors like the malaria mosquito *Anopheles sacharovi* Favre (Diptera: Culicidae), *Anopheles albimanus* Wiedemann (Diptera: Culicidae), and the house fly *Musca domestica* Linnaeus (Muscidae: Diptera) have also been resistant to 20, 21, and 65 different insecticidal compounds, respectively (APRD, 2023). Ninety percent of malaria-endemic countries have documented resistance in Anopheles mosquitoes to at least one class of insecticide, with 32% reporting resistance to pyrethroids, carbamates, organophosphates, and organochlorines that were recommended until 2016 (WHO, 2012). IR eliminates pest management alternatives and may lower agricultural profitability. The availability of new insect-challenging chemicals is becoming increasingly difficult because of rising costs for discovery, development, and registration, fueled partly by public concerns about environmental safety and human health (Van Leeuwen et al., 2020).

Resistance is an evolutionary phenomenon characterized by toxicodynamic and toxicokinetic changes in the physiology and biochemistry of resistant strains, resulting in shifts in penetration, activation, metabolism, transport, and excretion - altering the amount of toxin that reaches the target site (toxicokinetic mechanisms), and alterations to the pesticide target-site due to structural changes, knock-out, and amplify mechanisms (toxicodynamic mechanisms) (Kennedy and Tierney, 2012; Feyereisen et al., 2015).

Insecticide resistance mechanisms can be broadly classified as follows: 1) behavioral resistance, 2) fitness cost, 3) penetration resistance, 4) target-site resistance, 5) metabolic resistance, and 6) resistance-inducing operational parameters (Siddiqui et al., 2023). Insect pests overcome both the host plant defenses and the toxicity of insecticides to adapt and survive. Detoxification genes are essential for pests to withstand plant poisons and insecticides at the molecular level. Many research studies in arthropods have shown that microRNAs (miRNAs/miRs) play critical roles in physiological and developmental pathways such as metamorphosis, embryogenesis, molting, reproduction, immunity, wing development, and metabolism of plant toxins and insecticide resistance (Qiao et al., 2019). MiRNAs are abundant in the insects’ genomes and are important regulators of gene expression in response to xenobiotic stressors.

Non-coding RNAs (ncRNAs) are crucial for managing insecticide resistance and pest control (Etebari et al., 2015). Recently, miRNAs associated with resistance have been found to target detoxification genes. Along with their verified target genes, their regulatory roles in insecticide resistance and detoxification in various pests have also been established (Zhang et al., 2021). The growing body of information suggests that oxidative and other cellular stress influence miRNA expression. This review majorly summarised miRNAs associated with insecticide-resistant pests and their potential relevance in insect pest management. Information on the involvement of epitranscriptomic regulation, long non-coding RNAs, and the xenobiotic pathways regulating detoxification genes has also been reviewed.

### Non-coding RNAs

The ncRNAs, diverse RNA molecules, including ribosomal RNA (rRNA), transfer RNA (tRNA), small ncRNAs (sncRNAs), and long ncRNAs (lncRNAs), are categorized based on their length and intended function. Small interfering RNA (siRNA), small nuclear RNA (snRNA), and PIWI-interacting RNAs (piRNA) with fewer than 200 nucleotides are classed as sncRNAs. Their sizes range from 18 to 25 nucleotides for small RNAs like siRNAs and miRNAs and from 20 to 200 nucleotides for other small RNAs. On the other hand, ncRNAs of more than 200 nucleotides are classified as long non-coding RNAs (lncRNAs), which are found in practically all eukaryotic creatures (Mercer et al., 2009). All of these RNAs typically operate as transcriptional and translational regulators.

### MicroRNAs

**FIGURE 1:**
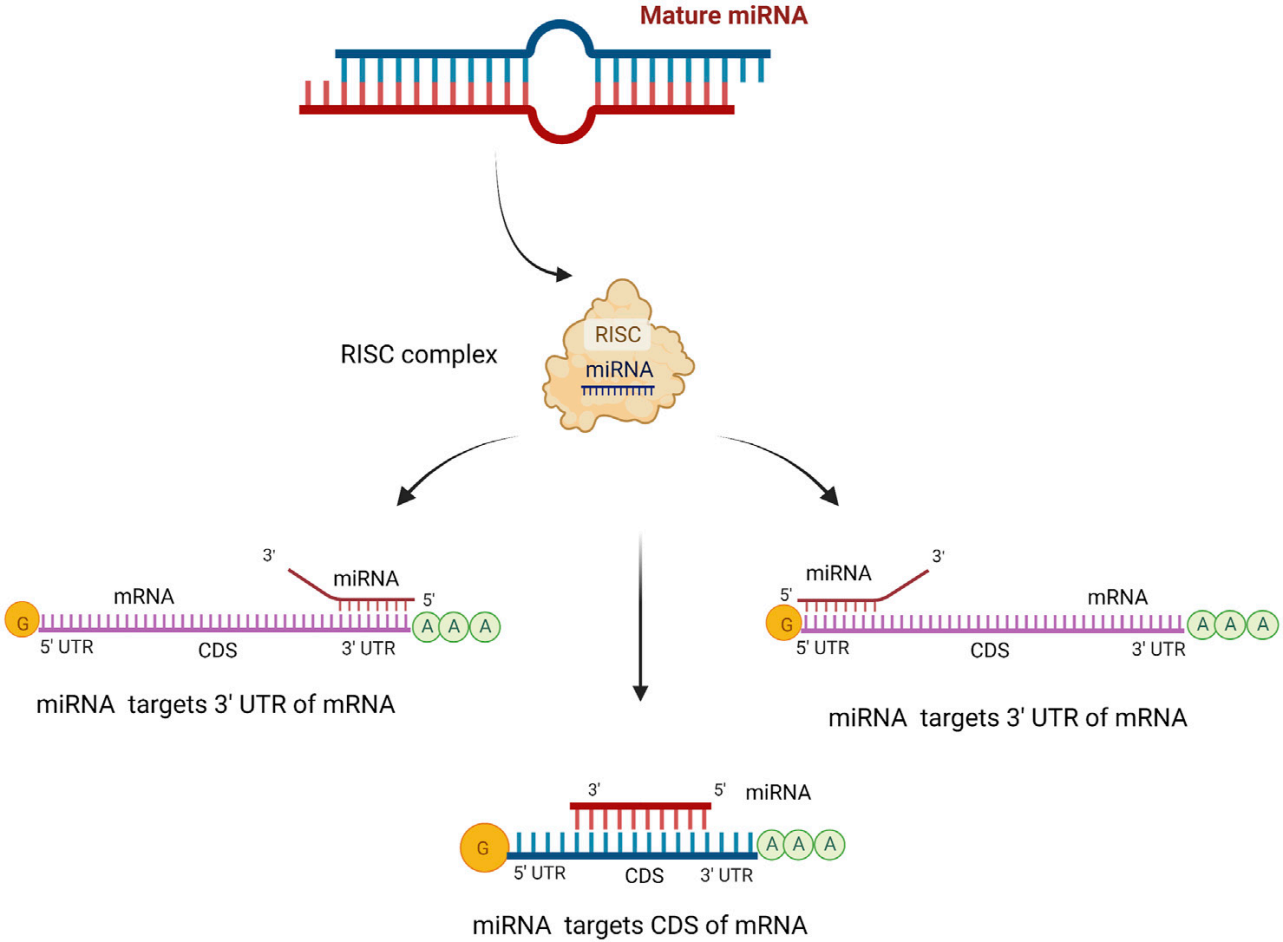
The target site of miRNA. MiRNAs can silence the expression of target genes by binding to 5′or 3′untranslated regions (UTRs) or coding sequence (CDS) of mRNA. MiRNA can bind with different complementarities, such as incomplete pairing in UTR regions and complete pairing in the CDS region. Created with BioRender.com.

MicroRNAs bind to the 3′-untranslated regions (UTR) of the messenger RNA of target genes via imperfect base pairing between the miRNA’s “seed” sequence (nucleotides 2-8 at its 5′end) and its complementary seed match sequence, causing post-transcriptional gene expression regulation. Initially, it was assumed that miRNA target sequences could only be found in the 3′UTR of target mRNAs. However, leads from research imply that target sequences may reside in the open reading frame, 5′UTR, and 3′UTR (Bartel, 2009) (Figure 1).

In a search for genes essential for post-embryonic development in the nematode *Caenorhabditis elegans* (Rhabditida: Rhabditidae), the first miRNA, lin-4, was found (Lee et al., 1993). Though, at the time, it was practically regarded as a genetic anomaly, the discovery of the lin-4 locus and its regulatory mechanism through the 3′UTR of lin-14 mRNA was intriguing. However, the identification of another miRNA, let-7, first in *C. elegans* (Reinhart et al., 2000) and then in many bilaterian species (Pasquinelli et al., 2000), proved that the interactions between lin-4 and lin-14 were not at all unusual but rather a new and fundamental layer of the mechanisms governing gene expression (Lai et al., 2003).

### MicroRNA biogenesis

MiRNAs may be encoded from non-coding transcripts, introns, or coding regions. MiRNA genes, mostly independent transcriptional units, are predominantly transcribed by RNA polymerase II as a primary miRNA (pri-miRNA), which may contain one or more stem loops. Like mRNAs, pri-miRNA transcripts are 5′capped and polyadenylated (Bracht et al., 2004). Drosha, associated with Pasha (equivalent to DGCR8 in mammals), further processes the stem loop into a short hairpin of around 70 bases known as the precursor miRNA (Bartel, 2009). Pre-miRNA hairpins may also be directly processed from primary mRNA transcripts by splicing and debranching of short introns, referred to as mirtrons. After being carried into the cytoplasm by Exportin-5, the terminal loop of pre-miRNA is removed by ribonuclease enzyme Dicer-1 (Dcr-1), resulting in a miRNA:miRNA* duplex with two nucleotide overhangs on both ends (Hutvagner et al., 2001). The duplex is integrated into the RNA Inducing Silencing Complex (RISC), which is mainly made up of the Argonaute-1 (Ago-1) protein (Miyoshi et al., 2009). The miRNA strand (guide strand) subsequently directs the RISC complex to the target mRNA once the miRNA* strand (passenger strand) has been torn and degraded.

### MicroRNA classification

MiRNA classification is based on the “seed sequence” region because selection pressures appear to regulate the nucleotide substitution pattern in miRNA genes and because it is a functionally important region (Brennecke et al., 2003; Bartel, 2009). In the case of metazoans, 858 miRNA families have been deposited in the miRBase database (v21.0) (Griffiths-Jones et al., 2007), with 254 (30%) of these families found in at least five species. These records may vary as more high-throughput sequencing experiments are performed; however, current statistics show that most miRNA families (562) are discovered in vertebrates, followed by insects (178 families recorded). Different miRNA families may have varying degrees of conservation in the seed region. The miRNAs miR-100, miR-125, and let-7 illustrate a well-conserved seed region (Behura, 2007). There are insect-specific miRNA families, including bantam, miR-2, and miR-3, whose seed region is likewise highly preserved.

### MicroRNA in insects

According to the InsectBase version 2.0 database, 112,162 miRNAs in 807 insects have been discovered (Mei et al., 2022). The insect with the highest number of miRNAs (576) is *Aedes albopictus* (Skuse) (Diptera: Culicidae). Insect development, including the formation of the germ cell, the wing, and the muscle, the neurogenesis, the apoptosis, and phenotypic plasticity, is greatly influenced by miRNA (Xu et al., 2003; Hilgers et al., 2010; Truscott et al., 2011; Ge et al., 2012; Li and Padgett, 2012). Specific miRNAs, including miR-263*,* miR-14*,* bantam, and the miR-2 family, have been discovered to affect apoptosis in *Drosophila melanogaster* Meigen (Diptera: Drosophilidae). Apoptosis is controlled by miR-263a/b as well. miR-8, a highly conserved miRNA, has been linked to the insulin signaling system in the fat body of *Drosophila* larvae (Hyun et al., 2009). The conserved miRNA miR-14 has been demonstrated to play a role in 20-hydroxyecdysone (20E) signaling pathway of *Drosophila*. The miR-14 mutant has a shortened lifespan because miR-14 participates in the 20E signaling pathway (Varghese and Cohen, 2007).

In insects, carbon dioxide (CO_2_) receptors are structured differently, resulting in various olfactory behaviors. Feeding-related behavior was correlated with olfactory detection of CO_2_ through neurons present in the mouthparts of an insect, such as maxillary palps (MPs) and labial palps. In the absence of miR-279 in *Drosophila*, a CO_2_ sensing system is developed in the maxillary palps, similar to that reported in mosquitoes (Cayirlioglu et al., 2008). miR-14, discovered in insulin-producing cells in the fly brain, regulates insulin production and metabolism in *Drosophila*, which explains why *miR-14* mutant flies are metabolically deficient (Varghese et al., 2010). miR-34 expression increases with age in the *Drosophila* brain (Liu N. et al., 2012), and miR-34 deficiency results in accelerated brain aging, late-onset brain degeneration, and lower survival, whereas miR-34 over-expression extends the median lifespan and reduces neurodegeneration. In *Drosophila*, miR-8 has been associated with neurodegeneration prevention (Karres et al., 2007). miR-7 functions within genes implicated in photoreceptor and proprioceptor determination in *Drosophila* to protect these networks from environmental changes and other pressures (Li et al., 2009).

MiRNAs miR-31a, let-7, miR-279, and miR-275 were over-expressed in the honey bee, *Apis mellifera* Linnaeus (Hymenoptera: Apidae) nurses than foragers, but miR-13b, miR-133, miR-210, miR-278, and miR-92a were downregulated in nurses compared to foragers (Liu F. et al., 2012). Putative miRNAs have been experimentally identified and mapped to the pea aphid genome. Two parthenogenetic pea aphid morphs, sexuparae and virginoparae, differed in their expression of five miRNAs: miR-34, miR-X47 and miR-X103, miR-307*, and miR-X52* (Legeai et al., 2010).

Although insects lack adaptive immunity, parts of their innate immunity involved in cellular and humoral responses can identify foreign things and then express the appropriate reaction to the foreign intruder in their presence. These include melanization, phagocytosis, nodule/capsule development, antimicrobial peptide synthesis, wound healing, and nodule/capsule formation (Lemaitre and Hoffmann, 2007). Due to their capacity to control gene expression at the post-transcriptional level, miRNAs may be essential for preserving the homeostasis and plasticity of immunity. For instance, it has been demonstrated that miR-8 negatively regulates the expression of antimicrobial peptides, such as Drosomycin and Diptericin, in *Drosophila* to keep their expression level low during typical non-infection settings, facilitating the homeostasis of immunity (Choi and Hyun, 2012).

### Databases related to microRNAs

In the post-genomic era, biological data are being created at an increasing rate with the development of high-throughput sequencing technologies (Katz et al., 2022). Using next-generation sequencing (NGS) technology, researchers could predict miRNAs from target insects more quickly and inexpensively. Experts worldwide established databases to globalize biological data, which might aid novice researchers in making quick and accurate discoveries on miRNAs. The primary databases with the bulk of information on insect miRNAs are InsectBase 2.0 and miRBase; additional useful databases are given in Table 1.

**TABLE 1:** Different databases related to miRNAs in insects.

Database	Information packed	References
miRBase	This database contains miRNA sequences, both mature and precursors of 31 insect species	Kozomara et al. (2019)
InsectBase2.0	This database contains many miRNAs (112,162) from 801 insect species	Mei et al. (2022)
ENA (European Nucleotide Archive)	It contains 2,661 miRNA sequences of more than 30 insects	Burgin et al. (2023)
MirGeneDB 2.1	9 insect species from four insect orders contains 1372 miRNAs	Fromm et al. (2020)
FlyBase	A database exclusively for *Drosophila* Genes and Genomes where it contains 440 miRNA sequences	Larkin et al. (2021)
BmncRNAdb	Database dedicated to *bombyx mori*, which contains 1,986 miRNAs	Zhou et al. (2016)

### MicroRNA regulation in insecticide metabolism

**FIGURE 2:**
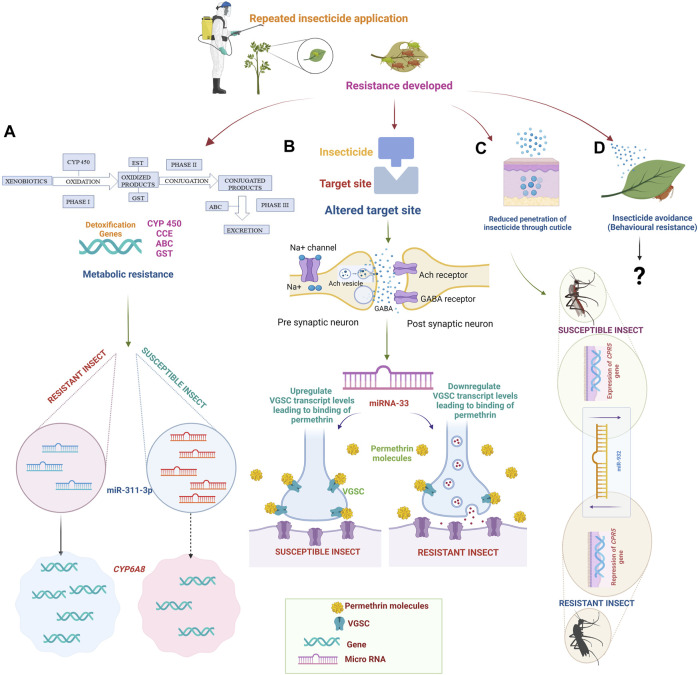
Mechanism of insecticide resistance. Due to the repeated application of insecticides, insects develop resistance. **(A)** Target site insensitivity: Mutations in the target receptors of insecticides. Nicotinic acetylcholine receptors (nAChRs), Gamma-aminobutyric acid (GABA), Voltage-gated sodium channel (VGSC), and Acetylcholine esterase (Ach) receptors may face resistance due to alterations in the structure or expression levels of these receptors. In permethrin susceptible insects, miR-33 upregulates the transcript level of VGSC, leading to increased binding of permethrin to VGSC. In the case of permethrin-resistant insects, the binding of permethrin to VGSC is less due to the downregulation of VGSC by miR-33. **(B)** Metabolic resistance: Insects possess a wide range of metabolic enzymes, such as cytochrome P450 monooxygenases (P450s), esterases, and glutathione S-transferases (GSTs), which can detoxify insecticides. miRNAs can bind to target detoxification gene mRNA molecules, leading to mRNA translational repression or degradation. The upregulation of miR-311-3p degrades the mRNA of the *CYP6AB* gene resulting in lower expression of *CYP6AB* in susceptible insects. In contrast, the resistant insects had overexpression of *CYP6AB* due to downregulation of miR-311-3p. **(C)** Reduced cuticular penetration: Insects possess a cuticle that acts as a barrier to the penetration of insecticides. Resistance can occur through the thickening or modification of the cuticle, reducing insecticide uptake. The *CPR5* gene has been highly expressed in susceptible insects due to the downregulation of miR-932. However, the *CPR5* gene is repressed in resistant insects due to the upregulation of miR-932. **(D)** Behavioral resistance: Some insects can develop behavioral changes that reduce their insecticide exposure. They may avoid treated areas, alter feeding or breeding behaviors, or exhibit reduced contact with insecticides. The involvement of microRNA in behavioral resistance has not been reported. Created with BioRender.com.

Four factors are primarily involved in the evolution of insect resistance to insecticides: increased metabolic capacity for detoxification, target insensitivity (Troczka et al., 2012), delayed cuticular penetration, and behavioral modification (Liu, 2007) (Figure 2).

According to most researchers, metabolic resistance is the primary mechanism underlying the early emergence of resistance (Hyun et al., 2009). Metabolic resistance is also intimately linked to the differential expression of genes encoding detoxification enzymes (Feng et al., 2018). Primary phase I, which entails hydrolysis or oxidation, and secondary phase II, which entails conjugating phase I products, are the two detoxification steps (Berenbaum and Johnson, 2015). Esterases (EST), monooxygenases (like those found in cytochrome P-450 (CYP)), and transferases (like glutathione-S-transferase (GST)) are only a few of the many enzymes that are crucial to its function. The overproduction of specific enzymes causes insecticides to degrade before binding to their target sites, and with these excessively generated enzymes, pests can develop resistance to insecticides. Notably, these enzymes degrade xenobiotics into non-toxic molecules. A list of studies describing insect microRNAs involved in detoxification of and resistance to different insecticides is reported in Supplementary Table S1.

### Lepidoptera

#### Diamondback moth, *Plutella xylostella*

The diamondback moth, *P. xylostella*, is a severe pest of crucifer crops such as cauliflower, mustard, radish, turnip, Chinese cabbage, broccoli, rape, and kale (Ganehiarachchi and Knodel, 2008). It has become one of the most resistant pests in the world due to the repeated application of insecticides. It has developed resistance against organophosphates, pyrethroids, new molecules, and microbial-derived pesticides (Furlong et al., 2013). To date, 1010 cases of insecticide resistance to more than 101 insecticides have been recorded due to dependence on insecticides to control diamondback moth (APRD, 2023). In *P. xylostella*, enhanced detoxifying enzyme activity (56%) and altered target sites (44%) are the two leading causes of different pesticide resistance (Banazeer et al., 2021). The pesticide resistance in *P. xylostella* has been demonstrated to be significantly influenced by miRNA. Recently, chlorantraniliprole has been one of the pesticides most frequently employed on *P. xylostella*. It is an anthranilic diamide insecticide that opens up the muscles’ ryanodine receptor (RyR) (Lahm et al., 2009). *Plutella xylostella* has developed resistance to chlorantraniliprole due to point mutations in the RyR gene and increased activity of detoxifying enzymes like CYPs, carboxylesterase (CarE), and GSTs (Liu et al., 2015).

**FIGURE 3:**
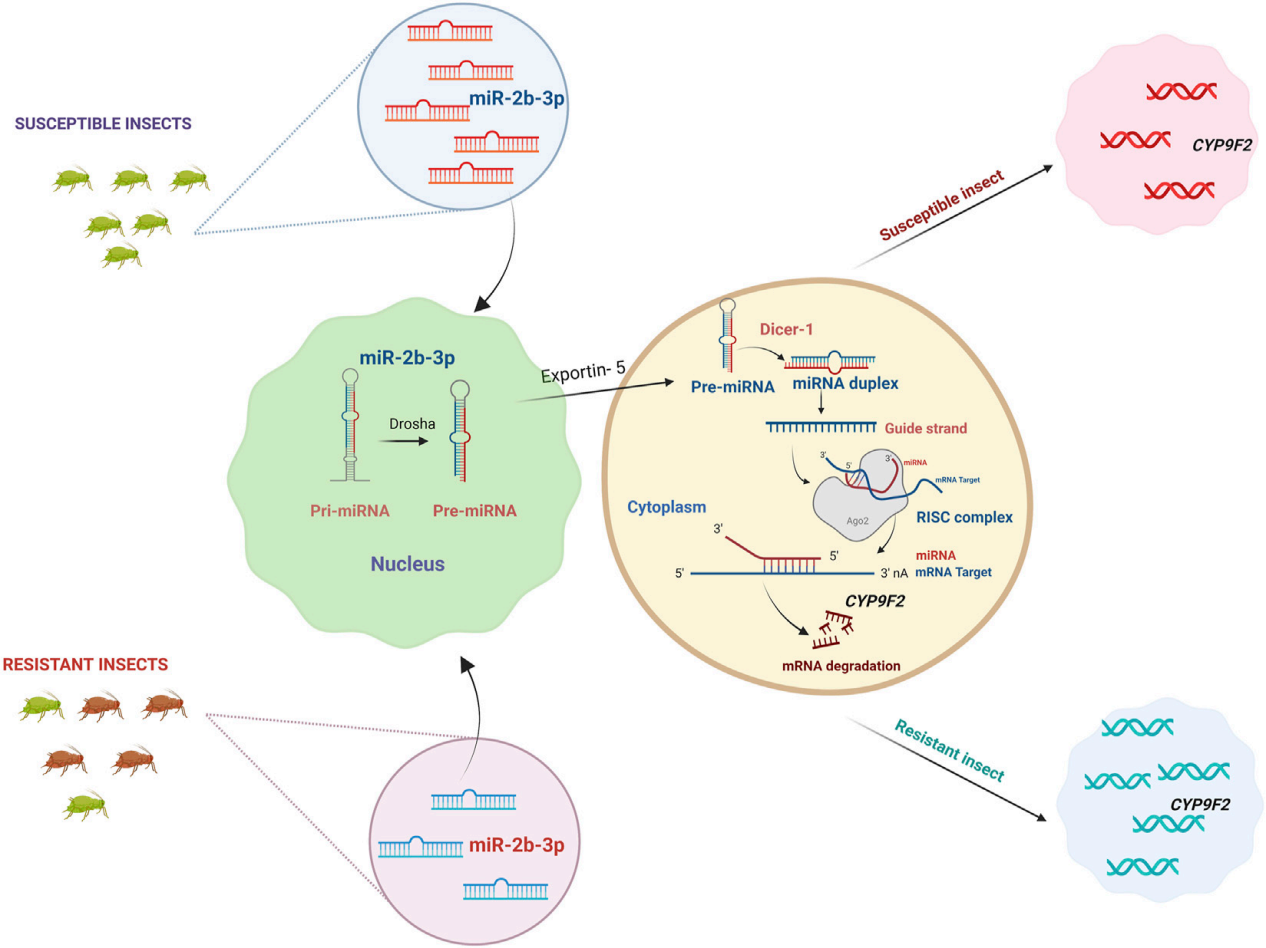
miRNAs regulating detoxification genes. miRNA genes are transcribed by RNA polymerase II to primary miRNA (pri-miRNA) with one or more stem-loops. Drosha further processes the stem loop into precursor miRNA. Pre-miRNA is carried into the cytoplasm by Exportin-5, and the terminal loop of pre-miRNA is removed by ribonuclease enzyme Dicer-1 (Dcr-1), resulting in a miRNA:miRNA* duplex. The duplex is integrated into the RNA Inducing Silencing Complex (RISC), mainly made up of the Argonaute-1 (Ago-1) protein. The miRNA strand (guide strand) subsequently directs the RISC complex to the target mRNA, and the miRNA* strand (passenger strand) will be degraded. miRNAs primarily bind to the mRNA of target molecules, leading to mRNA translational repression or degradation. They are involved in the post-transcriptional regulation of detoxification gene expression involved in insecticide metabolism, and the expression is negatively correlated between microRNAs and detoxification genes. In the case of susceptible insects, the microRNA miR-2b-3p is upregulated, where these microRNAs bind to the mRNA of detoxification gene *CYP9F2* and degrade them. The *CYP9F2* genes produced are fewer in number, which is not sufficient for insecticide detoxification, making the insect susceptible. In contrast to susceptible insects, miR-2b-3p is downregulated, which upregulates *CYP9F2* genes, and the insect creates resistance. Created with BioRender.com.

**FIGURE 4:**
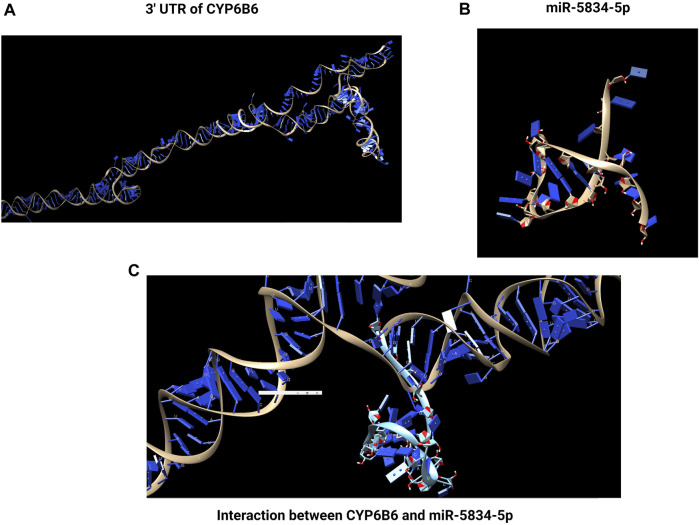
Interaction between miRNA and detoxification gene. The binding of a miRNA to its target site typically occurs in the 3′untranslated region (UTR) of the mRNA. In chlorantraniliprole exposed *to P. xylostella, miR-8534-5p* was downregulated, and its corresponding target *CYP6B6* was upregulated. **(A)** 3′ UTR of *CYP6B6*. **(B)** *miR-5834-5p*. **(C)** Interaction of *CYP6B6* and *miR-5834-5p*. Created with BioRender.com.

Detoxifying enzyme *CYP9F2* has a binding site for miR-2b-3p in its 3′UTR, but the binding sites for the other two miRNAs, miR-14b-5p and let-7-5p, were *CYP9F2* and *CYP307a1*, and *GST* and *CYP9F2*, respectively (Etebari et al., 2018) (Figure 3). Four common differentially expressed miRNAs (miR-8491-5p, miR-4969-5p, mir-8488-5p, and novel-13_1575) in chlorantraniliprole exposed *P. xylostella* two resistant and one susceptible strain. At the same time, miR-8533-3p, miR-8534-5p, and miR-375-5p were downregulated, and their corresponding targets included larval cuticle protein *LCP-30*, *CYP6B6*, and *CYP4G15*, respectively were upregulated after chlorantraniliprole exposure (Zhu et al., 2017) (Figure 4).

**FIGURE 5:**
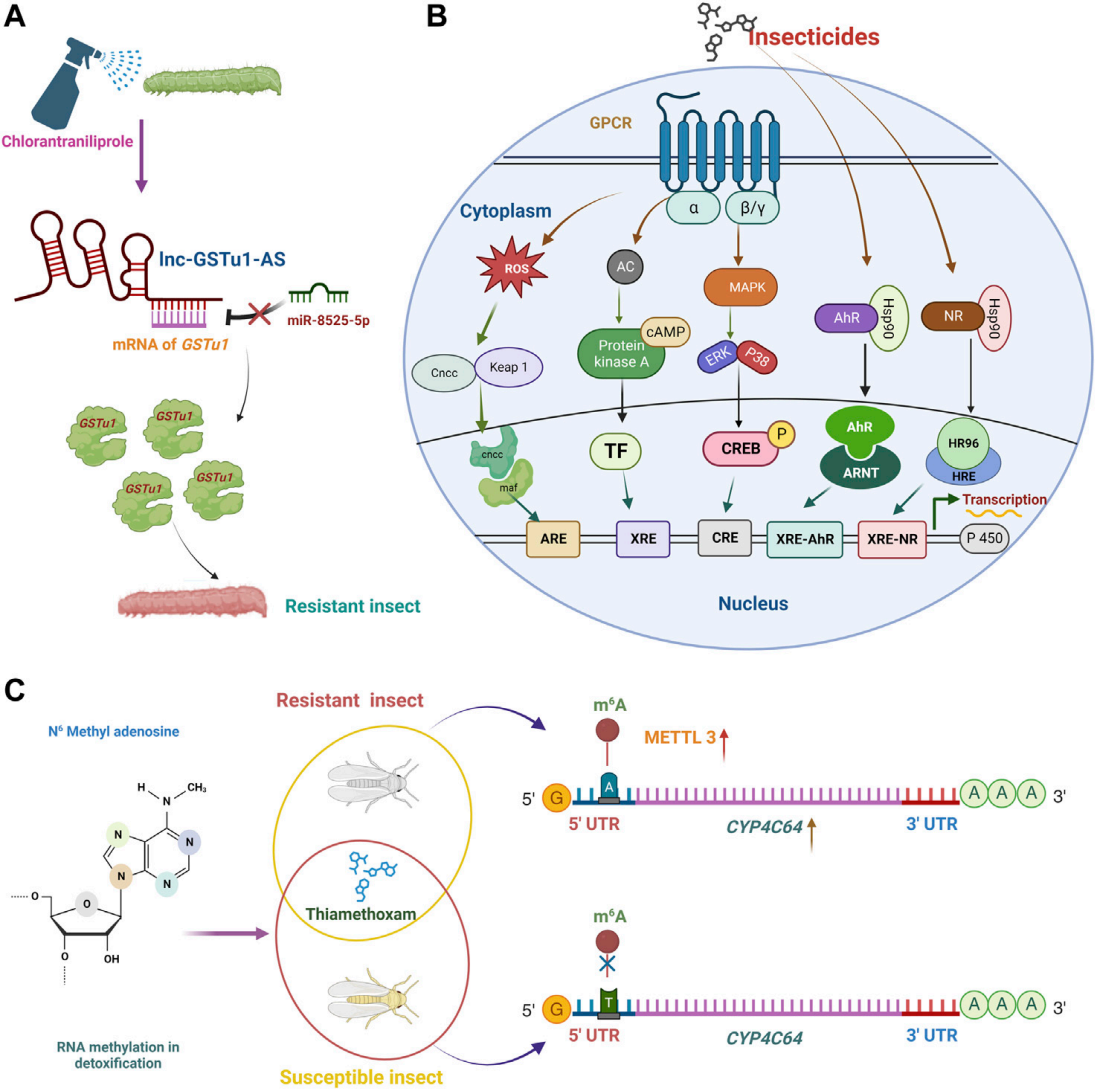
The regulation of insecticide resistance mechanisms involves epi-transcriptome signals, detoxification signaling pathways, and long non-coding RNAs (lncRNAs). **(A)** lncRNAs influence miRNA-mediated insecticide resistance regulation. Regulation mechanisms include lncRNA-miRNA-mRNA interaction and lncRNA-mediated regulation of miRNA expression. *GSTu1*, a detoxifying gene, is involved in the chlorantraniliprole resistance of *P. xylostella*. A long non-coding RNA, lnc-*GSTu1*-AS, interacted with *GSTu1* by forming an RNA duplex, masking the binding site of microRNA, miR-8525-5p, at the *GSTu1*-3′ UTR. lnc-*GSTu1*-AS maintained the mRNA stability of *GSTu1* by preventing its degradation, which could have been induced by miR-8525-5p and thus resulting in increased production of detoxification gene (*GSTu1*), increased the resistance of *P. xylostella* to chlorantraniliprole. **(B)** Detoxification enzyme induction pathway. This pathway involves upregulating detoxification enzymes, such as cytochrome P450 monooxygenases, esterases, and glutathione S-transferases. They are the CncC/Keap1, NR, PKA, MAPK/CREB, and AhR/ARNT pathways. The activated molecules of these pathways, such as Cncc/maf, TF, CREB, Ahr/AHRNT, and HR96/HRE, respectively, in the cytoplasm, interact with their corresponding response element in the nucleus to regulate the expression of detoxification genes through transcription. The arrows indicate the cascade of effectors in the signaling pathway. **(C)** N6-methyladenosine (m^6^A) is a modified form of adenosine widely involved in gene expression regulation. Mutation (T to A at position-206 bp) was observed in the 5′UTR of *CYP4C64* that was observed at a much greater frequency in the thiamethoxam-resistant strains compared with the susceptible strain. The T at 206 bp helps bind m^6^A, and the overexpression of the enzyme METTL (methyltransferase) led to the development of thiamethoxam-resistant insects. CncC, Cap‘n’ Collar isoform C; Maf, Musculoaponeurotic fibrosarcoma; ARE, Antioxidant responsive element; Gas, G protein alpha unit which stimulates adenyl cyclase; AC, adenyl cyclase; cAMP, cyclic adenosine monophosphate; PKA, protein kinase A; TF, Transcription factor; XRE, xenobiotic response element; MAPK, mitogen-activated protein kinase; ERK, extracellular regulated protein kinase; P38, P38 mitogen-activated protein kinase; CREB, c-AMP response element binding protein; -P, phosphorylation; CRE, cAMP response element; NR, nuclear receptor; AhR, aryl hydrocarbon receptor; Hsp90, heat shock protein 90; ARNT, aryl hydrocarbon receptor nuclear translocator; XRE-NR, xenobiotic response element-nuclear receptor; XRE-AhR, xenobiotic response element-aryl hydrocarbon receptor. Created with BioRender.com.

Seven possible miRNAs were predicted to target the *PxEcR-B* following fufenozide treatment. *Plutella xylostella* showed a 2.28-fold increase in the expression of miR-189942 and a 29% decrease in the expression of *PxEcR-B* (Li et al., 2020). The ATP-binding cassette transporter (ABC transporter) proteins have been recognized as essential receptors for several Cry toxins in insects. By interacting with the *ABCC2* gene’ coding sequence (CDS) in opposition to the *Cry1Ac* toxin, miR-998-3p enhanced Cry toxins resistance in *P. xylostella* (Zhu et al., 2020). Besides CYPs, GSTs also significantly impact *P. xylostella* ability to withstand chlorantraniliprole. For instance, *lnc-GSTu1-AS*, antisense transcript formed an RNA duplex with *GSTu1*, preventing miR-8525-5p from binding at the *GSTu1*-3′ UTR and therefore masked *GSTu1* degradation that could have been induced by miR-8525-5p and thus increased the resistance of *P. xylostella* to chlorantraniliprole (Zhu et al., 2021) (Figure 5A).

*ABCG20*, a member of the ABCG subfamily, was discovered to be substantially expressed in Cry1Ac-susceptible *P. xylostella* population, and its associated miRNA, a novel-miR-310, was predicted. Thirty-four miRNAs were discovered to have at least one binding site that targets the CDS of *ABCG20*. In contrast to the *ABCG20* expression pattern, novel-miR-310 was much more abundant in the Cry1S1000 strain (resistant) than the G88 strain (susceptible). A high-throughput sequencing analysis of small RNA libraries constructed from the midguts of the *P. xylostella Cry1Ac*-resistant strain and the *Cry1Ac*-susceptible strain revealed 12 differentially expressed miRNAs between the strains, with specific, nine miRNAs downregulated and three upregulated in the resistant strain. The discovered mRNA targets were genes involved in the cellular process, metabolism, membrane and catalytic activity, and the Hippo, MAPK signaling pathway (Yang et al., 2022).

The juvenile hormone esterase (JHE) gene *PxJHE*, whose inhibition increases *Cry1Ac* protoxin resistance, was differently expressed in the *Cry1Ac*-resistant and *Cry1Ac*-susceptible strains. Two novel miRNAs (miR-108 and miR-234) that were in inverse connection with the degree of *PxJHE* expression were predicted to target the *PxJHE* CDS (Yang et al., 2023).

#### Fall armyworm, *Spodoptera frugiperda*

The fall armyworm, *Spodoptera frugiperda* (Smith) (Lepidoptera: Noctuidae), is a critical migratory pest worldwide (Zhao et al., 2020). *Spodoptera frugiperda* is a severe maize pest with over 80 distinct crop hosts. Excessive pesticide use has resulted in resistance to 29 active insecticidal chemicals across six modes of action (Chao et al., 2019; APRD, 2023). Target site insensitivity is one of the reported mechanisms of insecticide resistance in *S. frugiperda*, such as the RyR that confers resistance to diamide insecticides (Boaventura et al., 2020), acetylcholinesterase (AChE) that confers resistance to carbamates and organophosphates, and voltage-gated sodium channel (VGSC) imparts resistance to synthetic pyrethroids (Carvalho et al., 2013). Resistance to pyrethroids, organophosphates, and carbamates is caused by metabolic detoxification, wherein chromosomal changes result in GST, CYP, and CarE gene amplification, over-expression, and alteration. Chlorantraniliprole significantly increased and decreased the expression of *CYP6K2* and miR-190-5p in *S. frugiperda* by 2.96-fold and 56.5%, respectively (Zhang M. Y. et al., 2022).

### Other Lepidopterans

The Asian spongy moth, *Lymantria dispar* (Linnaeus) (Lepidoptera: Erebidae), is a global forest pest that kills over 500 plant species (Zhang et al., 2023a). examined miRNA and mRNA levels in *L. dispar* larvae treated with cyantraniliprole, the anthranilic diamide insecticide of the second generation, used to combat Lepidopteran, Coleopteran, Dipteran, and Hemipteran pests (Liu, 2007; Foster et al., 2012). Eleven differently expressed miRNAs predicted twenty-one genes relevant to insecticide resistance, with 25 miRNA-mRNA interactions discovered. *CYP4C1* was the only differentially expressed gene in the miRNA-mRNA network influenced by novel-miR-4 upregulation (Zhang et al., 2023a). A lncRNA in cadherin allele intron 20 was recently found to modulate cadherin 1 transcription in the pink bollworm *Pectinophora gossypiella* (Saunders) (Lepidoptera: Gelechiidae). The lncRNA promotes *PgCad1* transcription and pink bollworm sensitivity to *Cry1Ac* (Li S. et al., 2019).

### Planthoppers

The brown planthopper (BPH), *Nilaparvata lugens* (Stal) (Hemiptera: Delphacidae), is a highly harmful pest in rice-growing parts of Asian countries (Hereward et al., 2020). Because of the extensive use of insecticides, BPH has developed high levels of resistance to the major classes of insecticides, covering 34 active components of pesticides, with 453 recorded resistance cases worldwide, including neonicotinoids, phenylpyrazoles, carbamates, pyridine azomethine derivatives, and inhibitors of chitin biosynthesis (Liao et al., 2021; APRD, 2023). Resistance mechanisms in BPH have included target-site mutation and upregulation of detoxifying enzyme genes (Bao and Zhang, 2019).

The ABC transporter was involved in *N. lugens* resisting nitenpyram and clothianidin. Fourteen and four ABC genes were considerably increased in nitenpyram- and clothianidin-resistant *N. lugens* strains, respectively, with *ABCD3* and *ABCG3* highly over-expressed in both nitenpyram and clothianidin-resistant strains. The novel_268 miRNA has been predicted to target the *ABCD3* and *ABCG3* CDS (Li et al., 2022). Furthermore (Mao et al., 2022), discovered 72 differently expressed miRNAs in *N. lugens*, with 29 miRNAs being over-expressed and 28 miRNAs being downregulated on exposure to nitenpyram. The bioinformatics study showed that novel 85 and novel 191 have been predicted to target the CDS of *CYP6ER1* and *CarE1*, respectively.

### Aphids

The Aphididae family contains around 4,000 species of aphids, although just 20 are highly polyphagous and infested plants from 50 families (Blackman and Eastop, 2000). Aphids are almost resistant to most commonly used insecticides such as organophosphates, carbamates, pyrethroids, neonicotinoids, and newer insecticides (Guo et al., 2017). miRNA influences aphid resistance development activities through i) post-translational modification of CYP genes, ii) downregulation of insecticide receptors, and iii) causing the over-expression of acetyl co A carboxylase, a crucial enzyme in fatty acid biosynthesis. Recent advances in next-generation sequencing (NGS) technologies have facilitated the identification of aphid miRNAs*. Acyrthosiphon pisum* (Harris) (Hemiptera: Aphididae) was the first aphid to have its miRNA sequenced and used as a reference database (Legeai et al., 2010).

MiRNA profiling of *M. persicae* revealed 22 miRNAs that actively bind to the target gene *CYP6CY3*, which modulates resistance to nicotine, the plant’s secondary metabolite. Among these 22 miRNAs, let-7 and miR-100 are linked to post-translational alteration of the *CYP6CY3* gene, which leads to enhanced nicotinic compound breakdown (Peng et al., 2016). Similarly, *Aphis gossypii* (Glover) (Hemiptera: Aphididae) adapts to gossypol, a plant toxin, and other secondary metabolites such as tannic acid by overexpressing CYP genes (Du et al., 2004; Peng et al., 2017). A novel CYP450 gene, *CYP4CJ1*, discovered in *A. gossypii*, was associated with the mechanism of gossypol resistance, and *CYP4CJ1* gene over-expression was governed by miR-4133-3p via reduced expression (Ma et al., 2019). Spirotetramat is a cyclic keto-enol insecticide targeting the enzyme acetyl-CoA carboxylase (ACC) essential for lipid biosynthesis (Nauen et al., 2008). Two miRNAs, miR-276 and miR-3016, were downregulated in the spirotetramat-resistant strain of *A. gossypii*, involving post-translational modification of the ACC gene (Wei et al., 2016).

In *Sitobion miscanthi* (Takahashi) (Hemiptera: Aphididae), downregulation of miR-278 and miR-263b is essential in targeting the nicotinic acetylcholine receptors *nAchRα1* and *nAchRβ1*, resulting in imidacloprid insensitivity. Also, the downregulation of miR-316 targets the over-expression of the *CYP4CJ6* gene, resulting in imidacloprid resistance (Zhang B. Z. et al., 2022).

#### Mite, *Tetranychus cinnabarinus*

Over 100 crops, including cotton and beans, are fed by the carmine spider mite, *Tetranychus cinnabarinus* (Boisduval) (Trombidiformes: Tetranychidae) (Jia et al., 2011). The primary method of mite management involves using insecticides and acaricides, which inevitably results in the development of resistance. Due to their high fecundity, short generation period, and high inbreeding tendency, mites are more predisposed to pesticide resistance problems than other crop pests. In New York in 1949, there was a first-ever report of parathion resistance in *T. cinnabarinus*. Variations in the activity of several detoxification enzymes, including MFO, GST, and CarE, are linked to acarid resistance. *Tetranychus cinnabarinus* developed cyflumetofen resistance in 2014, with significantly greater activity levels of the detoxifying enzymes CarE, CYPs, and GSTs (Wang et al., 2014).

The miR-1-3p (miR-1 family) levels were lower in cyflumetofen-resistant *T. cinnabarinus* and that the detoxifying enzyme gene *TCGSTM4* (mu class GST gene) was a target of miR-1-3p (Zhang et al., 2018). It was the first report of a miRNA and its target implicated in acaricide (cyflumetofen) resistance in *T. cinnabarinus*. Fenpropathrin is a broad-spectrum insecticide widely employed against mites and pests of many crops (Solomon et al., 2001). *Tetranychus cinnabarinus* resistance mechanism to fenpropathrin comprises a mutation in the sodium channel gene (F1538I), target site resistance, and increased enzyme activity of GST, CYP, and carboxyl choline esterase (CCEs). Using transcriptome sequencing, 4,454 lncRNAs in the carmine spider mite *T. cinnabarinus* were discovered (Feng et al., 2020). Among these, the detoxifying enzyme gene *TcGSTm02* and *lincRNA_Tc13743.2* each had a miRNA (miR-133-5p) response element. A cyflumetofen-resistant strain of *T. cinnabarinus* (CyR) was found to over-express *lincRNA_Tc13743.2* and *TcGSTm02*, while miR-133-5p was downregulated.

#### Mosquito, *Culex pipiens pallens*

Mosquitoes are vectors for various illnesses that can significantly affect human health. Mosquitoes must be managed effectively to prevent and control mosquito-borne illnesses. Excessive usage of pesticides against mosquitoes in recent decades has resulted in insecticide resistance, which impedes efficient control. Studies show that the targeted areas’ decreasing sensitivity has led to complex multiple insecticide resistance. miRNAs degrade target mRNAs and regulate host-pathogen interactions, metabolism, development, and pesticide resistance.

miRDeep prediction methods assisted in discovering nine novel miRNAs from *C. pipiens pallens* (Linnaeus) (Diptera: Culicidae). The most abundant among them is miR-13664, which was identified to target the 3′-UTR of *CYP314A1* (Sun et al., 2019). miR-932 was found to be 1.8-fold over-expressed, resulting in a 2.8-fold decrease of its target *CPR5* (Liu et al., 2016). In another instance, miR-4448 was 6.49-fold more abundant while its predicted target gene, *CYP4H31*, was 2.77-fold less abundant in the deltamethrin-susceptible strain (Li et al., 2021a). *CYP6N23*, *CYP6AG11*, *CYP9J35*, *CYP325BG3*, and *CYP6Cp1* were shown to be inversely linked with their respective miRNAs (miR-285, miR-278-3P, miR2, miR-71). *CYP9J35*, on the other hand, had a positive connection with its regulator miR-13 (Fahmy et al., 2020).

#### Fruit fly, *Drosophila melanogaster*

The dichloro diphenyl trichloroethane (DDT) resistance in the fruit fly, *D. melanogaster*, was due to a single CYP gene, *CYP6G1* (monogenic) in low-level DDT resistance phenotype (Daborn et al., 2001; Le Goff and Hilliou, 2017) but moderate to high-level DDT resistance was found to be polygenic due to multiple resistance genes (Kim et al., 2018). *Drosophila* metabolic resistance to DDT has been linked to constitutively over-expressed genes and higher levels of CYPs (P450s), GSTs, and ESTs (Tu and Akgül, 2005). The 3′UTR sequences of DDT-resistant *D. melanogaster* over-expressed genes *CYP6G1*, *CYP6G2*, *CYP6A8*, and *CYP4G1* had a target site for their respective regulator miRNAs miR-310-3p, miR-311-3p, miR-312-3p, miR-313-3p, and miR-92a-3p, which exhibited downregulation following DDT exposure (Seong et al., 2019).

### Long non-coding RNAs

Although lncRNAs are not translated into proteins, they have a similar structure to mRNA. The lncRNAs, previously considered insignificant, are increasingly garnering attention due to their regulatory involvement in various biological processes in animals and plants. The lncRNAs have a role in the stability and translation of mRNAs, pre-mRNA splicing, and protein activities and also serve as precursors of siRNA and miRNA in post-transcriptional control. Based on InsectBase version 2.0 database, 1,293,430 lncRNAs in 376 insects have been discovered (Mei et al., 2022). According to the region of the genome that was transcribed, lncRNAs are divided into four categories: 1) sense lncRNAs overlap exonic regions of another transcript made from the same strand; 2) antisense lncRNAs are present on the complementary strand of the sense strand; 3) intergenic lncRNAs (lincRNAs), which are made from the DNA between two genes (intergenic regions); 4) bidirectional lncRNAs are concurrently transcribed at the opposing strands from coding transcripts (Quinn and Chang, 2016). Recent research has demonstrated the importance of lncRNAs in all aspects of insect development, reproduction, and genetic plasticity (Choudhary et al., 2021). According to recent studies, lncRNAs have been linked to enhanced fitness, xenobiotic sensitivity, and pesticide resistance (Lawrie et al., 2022).

Researchers discovered 6,171 lncRNA transcripts from *Bactrocera dorsalis* (Hendel) (Diptera: Tephritidae) malathion-resistant (MR1) and susceptible (MS) strains, including 3,728 lincRNAs, 653 antisense lncRNAs, 1,402 intronic lncRNAs, and 388 sense lncRNAs. Twenty-seven of these lncRNAa were expressed similarly in both males and females of the MR1 strain, with only 15 lncRNAs upregulated and 12 downregulated. Among them, the MR1 strain cuticle showed significant levels of expression of the lncRNAs *lnc15010.10* and *lnc3774.2*, indicating that these two lncRNAs may be related to malathion resistance (Meng et al., 2021).

Differential gene expression patterns of lncRNAs in *A. gossypii* revealed 6,059 lncRNAs in spirotetramat-resistant (SR) and susceptible (SS) strains. Among them, 874 lncRNAs were differentially expressed, of which *MSTRG.28822.1*, *MSTRG.28822.2*, *MSTRG.28822.3*, *MSTRG.28822.4*, and *MSTRG.28822.5*, were predicted to be acetyl-CoA carboxylase (ACC) targeting. A combined study of reverse transcription real-time quantitative PCR (RT-qPCR) and RNA interference (RNAi) confirmed that the selected ACC lncRNA was related to the ACC expression it was predicted found that the transcription factors, *C/EBP* and *C/EBPzeta* were regulating ACC lncRNA (Peng et al., 2021).

Furthermore, when the *CYP380C6*, *CYP4CJ1*, *CYP6DA2*, *CYP6CY7*, and *CYP6CY21* genes which were discovered to be important for spirotetramat resistance in *A. gossypii* SR strain, when were ectopically expressed in *Drosophila* resulted in significantly decreased mortality after spirotetramat exposure. Silencing investigations revealed that lncRNAs *MSTRG.36649.2/5* and *MSTRG.71880.1* influence *CYP6CY21* and *CYP380C6* expression, affecting the sensitivity of the SR strain to spirotetramat (Peng et al., 2022).

Totally 11,978 lncRNAs, including 3,136 intergenic lncRNAs, 7,393 intronic lncRNAs, and 1,449 antisense lncRNAs, were identified from indoxacarb susceptible (SS) and resistant strains (Lab-InRS and Field-FInRS) of *Spodoptera litura* (Fabricius) (Lepidoptera: Noctuidae). Compared with the SS, 51 and 134 lncRNAs were upregulated and downregulated in the two resistant strains, respectively, and 908 differentially expressed mRNAs were their target genes. Expression of 14 P450s, seven CCEs, one GST, six UGTs, five ABC transporters, and 24 cuticle protein genes was under 112 differentially expressed lncRNAs. By sponging 10 miRNAs, 79 differentially expressed lncRNAs controlled the expression of 14 detoxifying and 19 cuticle protein genes that lead to indoxacarb resistance. The regulatory pathways of lncRNA-mRNA and lncRNA-miRNA-mRNA resulted in indoxacarb resistance with 47 differentially expressed lncRNAs. The involvement of *LNC_004867* and *LNC_006576* in *S. litura* indoxacarb resistance was confirmed through molecular and bioassay studies (Shi et al., 2022).

### Mutations

In nitenpyram-resistant (NitR) and imidacloprid-resistant (ImiR) strains of *N*. *lugens*, the transcription factor FoxO controlled *CYP4CE1* expression. In the differential influence of FoxO on *CYP4CE1* expression, *CYP4CE1* promoter sequence variations between susceptible and resistant insects were discovered. Single-nucleotide polymorphisms (SNPs) in six FoxO response locations predicted in the *CYP4CE1* promoter were found in more than 50% of NitR and ImiR strains. The active control of *CYP4CE1* expression by FoxO was primarily caused by two mutations, −650T/G and −2205T/A, in two response elements located at −648 bp and −2,200 bp, respectively (Zhang H. et al., 2023).

The *GSTe2* polymorphism analysis discovered a strong link between the point mutation and DDT resistance in the malaria vector *Anopheles funestus*, Giles (Diptera: Culicidae) with a single amino acid substitution L119F in the upregulated *GSTe* gene provided significant levels of metabolic resistance (Riveron et al., 2014).

*PxABCG*1 (*Pxwhite*), an ABC transporter gene, is implicated in the downregulation of *P. xylostella* Bt Cry1Ac toxin-functional midgut receptor, and the gene expression has been shown to be transcriptionally controlled. It was found that Antennapedia (Antp), a Hox family transcription factor, interacted with a cis-response element (CRE) in the *PxABCG1* promoter of the susceptible strain to stimulate gene expression. A cis-acting mutation, on the other hand, inhibited Antp from binding to the CRE and regulating the *PxABCG1* gene creating *Cry1Ac* resistance (Qin et al., 2021).

There were seven base alterations (M1-M7) in the *CYP6B7* promoter associated with resistance between fenvalerate-resistant (HDTJFR) and susceptible (HDTJ) strains of *H. armigera* (Hubner) (Lepidoptera: Noctuidae). pGL3-CYP6B7 reporter genes with varied mutation sites revealed that genes with M3, M4, and M7 mutations had significantly lower fenvalerate-induced activity. The transcription factors *Ubx* and *Br* were over-expressed in HDTJFR, with binding sites containing M3 and M7, respectively, confirming their role in fenvalerate resistance in *Helicoverpa armigera* (Huang et al., 2023).

Cloning and characterization of the *CYP332A1* gene linked with fenvalerate resistance in *H. armigera* revealed that the gene was more expressed in the resistant (BJ) strain than in the susceptible (HDS) strain. The resistant strain’s sequence had five amino acid changes (F21C, G28R, K64Q, A290S, and V477I). The 5′-flanking region of *CYP332A1* had potential binding sites for the transcription factors Dr, Ubx, Cf2, caup, ara, Antp, ftz, eve, and otp, and the resistant BJ strain had three significant deletions of 386–392 bp, 547–554 bp, and 965–971 bp (Huang et al., 2022).

*ACC* gene was significantly over-expressed in the spirotetramat-resistant strain compared to the laboratory-selected resistant and susceptible strains of *A. gossypii*. The full-length *ACC* gene sequenced from resistant and susceptible cotton aphids indicated a substantial relationship between spirotetramat resistance and 14 amino acid alterations in the ACC gene’s biotin carboxylase domain and carboxyl transferase domain (Pan et al., 2017).

### Copy number variation of detoxification genes and insecticide resistance

A specific DNA sequence copies vary in number among individual genomes, which is referred to as copy number variation (CNV). CNV in insect genomes is a rich source of potentially adaptive polymorphism, which may assist in overcoming the restrictions of purifying selection on conserved genes and allow for increased transcription (Weetman et al., 2018). CNV of detoxification genes such as GSTs, CYPs, ESTs, UGTs, and oxidative stress genes have been found in insects such as mosquitoes, tobacco cutworm, and fall armyworm (*S. frugiperda*). Adaptive evolution of multi-copy detoxifying genes has been implicated in insecticide resistance. Considerable allelic differentiation of genomic copy number changes between fall armyworm *S. frugiperda* regional populations but not among host-plant-based strains.

Insecticides containing organophosphates and carbamates, widely utilized to reduce mosquito populations worldwide, resulted in resistance to both insecticides and are conferred by the identical amino acid alteration (G119S) in the ace-1 gene. G119S mutation is part of homogenous duplications that associate multiple resistant copies of the ace-1 gene *in Anopheles gambiae* and *C. pipiens* (Gimenez et al., 2020). Multiple copies provided higher degrees of resistance, demonstrating the adaptability of the genetic architecture of resistance to organophosphate and carbamate insecticides surrounding the ace-1 locus (Milesi et al., 2022). Because of an increased gene copy number, overexpression of the *CYP6CY3* gene gives neonicotinoid resistance in the aphid *M. persicae* (Kirkland et al., 2023). Copy number variation increased *CYP6G1* gene expression in *D. melanogaster* (Schmidt et al., 2010). In brown planthopper *N. lugens*, the *CYP6ER1* gene had been duplicated, and allelic variations of some of the duplicated genes encoded enzymes that may metabolize imidacloprid (Heckel, 2021).

### Regulation of the expression of detoxification genes in insects

As inducing agents and substrates, xenobiotics are known to cause the over-expression of broad groups of genes involved in detoxification. Transcriptional regulation is frequently driven by cis-regulatory elements (cis-acting), which are short sequences within the promoter region that certain transcription factors bind (trans-acting) to and further recruit the transcriptional machinery (Guo et al., 2018). Several insect transcription regulatory pathways, such as AhR/ARNT, HR96, ROS/CncC/Keap1, GPCR/PKA, and MAPK/CREB pathways, govern insecticide and phytochemical detoxification (Figure 5B). A list of studies describing different transcription factors regulated by these pathways is reported in Supplementary Table S2.

### AhR/ARNT pathway

The xanthotoxin cascade is one of the first regulatory networks for detoxifying genes revealed in insects (Sogawa et al., 1995). They are found in insects and mammals and have bHLH DNA binding domains, Per-ARNT-Sim (PAS) protein-protein interaction domains, and ligand-binding domains (Denison et al., 1988; Hahn, 2002). In the cytoplasm, the molecular chaperone heat shock protein 90 (Hsp90) detects the inactivated forms of the Aryl hydrocarbon Receptor (AhR), the xenobiotic sensor. Following exposure to and binding to a variety of ligands, such as toxic substances, AhR is activated and translocated to the nucleus, where it heterodimerizes with ARNT (Aryl Hydrocarbon Receptor Nuclear Translocator) to affect the expression of numerous genes interacting with Xenobiotic Response Elements to AhR (XRE-Ahr) (Nakata et al., 2006).

*NlAhR* and *NlARNT* bound the *NlCarE7* promoter, significantly enhancing the transcriptional activity in *N. lugens* resistant to imidacloprid, etofenprox, and sporocarp. In *Locusta migratoria* (Orthoptera: Acrididae), *AhR* is associated with chlorpyrifos susceptibility by regulating *LmGSTd7* expression (Zhang et al., 2019).

In the cotton aphid *A. gossypii*, *CYP6DA2* is linked to gossypol and spirotetramat tolerance. In resistant strains of *A. gossypii*, AhR transcript levels were 9-fold more significant than in susceptible strains (Peng et al., 2017). Similarly, AhR/ARNT pathway was also engaged in nicotine tolerance in *M. persicae* via over-expression of *CYP6CY3* and *CYP6CY*4 (Pan et al., 2019). It was fascinating to learn that *CYP6CY3* is controlled by microRNAs, namely, let-7 and miR-100 (Peng et al., 2016). Transcription beginning point in the *CYP6B6a* promoter contained a short region of 138 bp that was highly connected to promoter activity in 2-tridecanone exposed *H. armigera* larvae (Li F. et al., 2014). This sequence (5′ -CATGACACCTG-3′) was comparable to Xenobiotic Response Element (XRE), suggesting that 2-tridecanone regulation of *CYP6B6* was mediated through the AhR/ARNT pathway. In chlorantraniliprole-exposed *P. xylostella*, the gene *CYP6B6*, was discovered to be controlled by miR-8534-5p (Zhu et al., 2017). Different pathways regulating detoxification genes and miRNAs are represented in Table 2.

**TABLE 2:** Pathways mediating in regulation of insect xenobiotic detoxification genes and microRNAs.

Detoxification enzyme	Reported insecticide	Reported insect	Pathway	miRNA associated	Study source	
					Target enzyme	miRNA
*CYP6CY3*	Nicotine	*Myzus persicae*	AhR/ARNT	let 7 miR 100	Pan et al. (2019)	Peng et al. (2016)
*CYP6B6*	2-tridecanone	*Helicoverpa armigera Plutella xylostella*	AhR/ARNT	miR-8534-5p	Li et al. (2014a)	Zhu et al. (2017)
*CYP6ER1*	Imidacloprid	*Nilaparvata lugens*	CncC Pathway	Novel_85	Tang et al. (2020)	Mao et al. (2022)
	Nitenpyram			Novel_191		
*CYP6G1*	DDT	*Drosophila melanogaster*	HR96 pathway	miR-310-3p	Cheesman et al. (2013)	Seong et al. (2019)
*CYP6A8*	DDT	*D. melanogaster*	CncC Pathway	miR-312-3p	Misra et al. (2013)	Seong et al. (2019)

### NR (Nuclear receptors) pathway

Three insect NRs, including HR96 (hormone receptor-like in 96), Hnf4 (Hepatocyte nuclear factor 4), and Ftz-f1 (Ftz transcription factor 1), have been implicated in phytochemical and insecticide regulation of P450s (Li et al., 2021b).

### HR96 (Hormone receptor-like in 96)

Hormone receptor-like in 96 (HR96) is the nuclear receptor superfamily (NR) transcription factor. The DNA binding domain (DBD), which consists of two zinc fingers, is a highly conserved functional domain in nuclear receptors. The ligand binding domain (LBD), which forms a ligand binding pocket, dimerization unit, and transactivation domain, is a less conserved functional domain (Germain et al., 2006; Markov and Laudet, 2011).

When activated, the two primary xenobiotic-binding nuclear receptors (NRs), constitutive androstane receptor (CAR), and Steroid and xenobiotic receptor/Pregnane X Receptor (SXR/PXR), translocate to the nucleus and dimerize with the retinoid-X receptor (RXR) to promote detoxification gene transcription. The ortholog HR96 gene in invertebrates represents CAR/PXR. Most arthropod genomes have it, including *T. urticae* (Grbic et al., 2011), *D. melanogaster* (King-Jones et al., 2006), *Tribolium castaneum* (Herbst) (Coleoptera: Tenebrionidae) (Xu et al., 2010), *A. mellifera* (Velarde et al., 2006), and *S. frugiperda* (Giraudo et al., 2015). Furthermore, it has been proposed that HR96 acts as a dimerizing partner of ultraspiracle protein (USP), the closest insect RXR orthologue, and as a dimerizing partner of insect ecdysone receptors (EcR) in mediating transcriptional regulation of the insect detoxification process (Giraudo et al., 2015).

In *Drosophila*, DHR96 is a crucial mediator of xenobiotic tolerance. The binding motif of DHR96 was found multiple times in the promoter region of two detoxification genes, *GSTe1* and *CYP6G1.* DDT is metabolized by *CYP6G1* via the HR96 route (Cheesman et al., 2013). Surprisingly, miR-310-3p affected the gene *CYP6G1* in *D. melanogaster* (Seong et al., 2019). *TcHR96* (*Tribolium HR96*) over-expression caused by imidacloprid detoxification considerably boosted the promoter activity of genes such as *CYP4Q4*, *CYP4G7*, *CYP4BR3*, and *CYP345A1* (Kim et al., 2021).

### HNF4 (Hepatocyte nuclear factor)

HNF4a, a member of the NR2A subfamily, acts as an important transactivator of P450 genes involved in drug metabolism and clearance (Jover et al., 2001). A single HNF4a ortholog called HNF4 is widely expressed in insect species and plays a role in diverse processes, including lipid mobilization, b-oxidation, insulin signaling, glucose homeostasis, and metabolic control. An imidacloprid-resistant strain (Res) exhibited a 251.69-fold resistance to imidacloprid compared to the susceptible counterpart (Sus). The expression level of Hepatocyte nuclear factor 4 (HNF4) in the Res strain was lower than that in Sus. When the *HNF4* was silenced*, UGT-1-7*, *UGT-2B10*, and *CYP6ER1* were significantly higher in the Res strain than in the Sus strain by 1.73-, 1.63-, and 4.94-fold, respectively. So, it was apparent that imidacloprid-resistant *N. lugens* was negatively regulated by *HNF4* (Cheng et al., 2021).

### FTZ (Fushi Tarazu)

Upregulation of *CYP6BG1* was responsible for chlorantraniliprole resistance *in P. xylostella.* The transcriptional factor FTZ-F1, an orphan nuclear receptor that binds to the FTZ gene, was found to regulate the transcriptional activity of *CYP6BG1.* Chlorantraniliprole potentiated the expression levels of *FTZ-F1* and *CYP6BG1* and was significantly higher in the resistant populations (Li X. et al., 2019).

### CncC/Keap1 pathway

The Cap'n'collar isoform C/Kelch-like ECH associated protein 1 (CncC/Keap1) pathway, a “master regulator” of gene transcription coding for enzymes, was shown to be involved in response to xenobiotic and oxidative stress. CncC is an ortholog of mammalian NF-E2-Related Factor 2 (Nrf2), a transcription factor in the basic leucine zipper (bZIP) family (Suzuki and Yamamoto, 2015). CncC(Nrf2) is extensively expressed and rapidly destroyed by the action of Keap1 under normal physiological circumstances. Nonetheless, during oxidative stress, ROS (reactive oxygen species) modify the CncC (Nrf2)/Keap1 complex, preventing CncC (Nrf2) breakdown. CncC interacts with Muscle aponeurosis fibromatosis (Maf) to promote the expression of genes with Antioxidant Response Elements (AREs) motif in their upstream region, such as numerous P450s and GSTs coding genes, which increases cellular ROS levels.

Temporal expression profiles of *B. dorsalis* revealed that the transcription factor *MafB* and detoxification genes were strongly expressed in the fat body and that abamectin stimulated the expression of *MafB, GSTz2*, and *CYP473A3* (Tang et al., 2019). Upstream sequence study of chlorpyrifos and cypermethrin-resistant *Spodoptera exigua* (Hubner) (Noctuidae: Lepidoptera) revealed that three GSTs (*SeGSTo2*, *SeGSTe6*, and *SeGSTd3*) have the same CncC/Maf binding site, while *SeGSTo2* and *SeGST6* share the AhR/ARNT binding site. In the presence of CncC and Maf proteins, luciferase activity driven by the *GSTe6* promoter was raised, and the presence of AhR and ARNT also boosted the transcriptional activity of the *GSTe6* promoter (Hu et al., 2019).

The *SlituCncC* gene of *S. litura* was shown to be more abundant in the Malpighian tubules, fat body, and midgut tissues of third and fourth instar larvae exposed to indoxacarb resistant strains than susceptible strains. When *SlituCncC* was knocked down in *S. litura*, 842 genes were downregulated, and 127 were upregulated. Six of these downregulated genes (*CYP367A1*, *CYP367B1*, *CYP341B21*, *CYP340L2*, *SlituCXE1*, and *SlituABCH-1*) were related with indoxacarb resistance in *S. litura* (*CYP367A1*, *CYP367B1*, *CYP341B21*, *CYP340L2*, *SlituCXE1*, and *SlituABCH-1*). The promoter regions of these six genes were predicted for the presence of CncC-Maf binding sites (Shi et al., 2021).

The CncC/Keap1 pathway was constitutively activated in two DDT-resistant *Drosophila* strains (RDDTR and 91R), together with over-expression of genes encoding possible DDT-detoxifying enzymes such as *GSTD1, CYP6A2*, and *CYP6A8* (Misra et al., 2013). Among these detoxifying enzymes, miR-312-3p controlled the gene *CYP6A8* (Seong et al., 2019). The imidacloprid-resistant *N. lugens* exhibits constitutive adipokinetic hormone (AKH) downregulation, which results in co-over-expression of CncC, Maf, and *CYP6ER1* (Tang et al., 2020). The enzyme *CYP6ER1*, which was shown to be implicated in neonicotinoid, Nitenpyram resistant strains, was controlled by two new miRNAs, Novel_85 and Novel_191, which may be mediated through the CncC pathway (Mao et al., 2022).

### MAPK/CREB pathway

The MAPK (Mitogen-Activated Protein Kinase) pathway is an evolutionarily conserved signaling system vital in many cellular functions in insects and other species. This system regulates growth, development, immunity, and responsiveness to environmental stimuli by transducing extracellular signals into intracellular responses. Extracellular ligands or signals, such as growth factors or cytokines, activate the route by attaching to their corresponding cell surface receptors. These receptors may be classified as receptor tyrosine kinases (RTKs) or G protein-coupled receptors (GPCRs). When ligands bind to the receptors, they alter conformation and activate intracellular signaling molecules. The renin-angiotensin system (RAS), a small GTPase that becomes activated by exchanging GDP for GTP, is one of the major molecules involved. Activated RAS then promotes the translocation of Rapidly Accelerated Fibrosarcoma (RAF), a serine/threonine protein kinase, to the cell membrane and induces conformational changes that lead to its activation. RAF then phosphorylates MEK (MAPK/Extracellular Signal-Regulated Kinase (ERK) Kinase), another serine/threonine kinase. MEK phosphorylates and activates ERK, also known as MAPK, the pathway’s terminal kinase. Active ERK enters the nucleus and phosphorylates different transcription factors, causing changes in gene expression. Depending on the environment, changes in gene expression generated by activated ERK result in a variety of biological responses. Cell proliferation and differentiation are examples of such reactions. Cell proliferation, differentiation, apoptosis, and immune response regulation are examples of such responses (Cruz et al., 2020). Recent research on whitefly shows that the MAPK/CREB signaling branch directly regulates *Bemisia tabaci* (Gennadius) (Hemiptera: Aleyrodidae) *CYP6CM1* expression, which assists in imidacloprid detoxification (Yang et al., 2020).

### MAPK pathway regulating Bt resistance in insects

*Bacillus thuringiensis* (Bt) is a Gram-positive, spore-forming bacteria capable of producing crystal proteins (Cry) that are poisonous to a broad range of insect species, mainly those of the orders Lepidoptera, Coleoptera, and Diptera. Bt also generates vegetative insecticidal protein (Vip) and secretory insecticidal protein (Sip), both of which are biodegradable and mainly target Coleoptera and Lepidoptera (Chakroun et al., 2016). Bt is highly particular in its activity and is extensively encouraged for pest management as an alternative to toxic chemical treatments. Nonetheless, insects have lately evolved resistance to Bt. Insect resistance to Cry toxins is thought to be caused by downregulation or mutation of the midgut proteinase and receptors, which results in decreased conversion of the protoxin to the active toxin and reduced toxin binding of Cry toxin to receptors (Ferre and Van Rie, 2002; Pigott and Ellar, 2007). Phenotypic association experiments and molecular expression analyses have revealed the required evidence for receptor-mediated Bt resistance developments. Currently, more than four types of functional receptors, including cadherin (Xu et al., 2005), aminopeptidase N (APN) (Tiewsiri and Wang, 2011), alkaline phosphatase (ALP) (Jurat-Fuentes and Adang, 2007), ATP binding cassettes (ABC) transporters (Atsumi et al., 2012; Xiao et al., 2014), and others, have been identified and validated in lepidopteran species related to the mode of action of Cry toxin action. The differential expression of the midgut membrane-bound ALP and ABC subfamily C (ABCC) genes have been implicated in high-level resistance to *Cry1Ac* in *P. xylostella*, and the connection between these genes is found to be regulated by MAPK cascades. The downregulation of *ALP*, *ABCC2*, and *ABCC3* was strongly correlated with recessive *Cry1Ac* resistance but not with the upregulation of *ABCC1*. While inhibiting *MAP4K4*, a constitutively transcriptionally-activated MAPK upstream gene within the *BtR-1* locus, led to a brief recovery of gene expression and restored the susceptibility in resistant larvae. However, silencing *ABCC2* and *ABCC3* in susceptible larvae reduced *Cry1Ac* susceptibility while having no effect on ALP expression (Guo et al., 2015).

MAPK cascade genes such as *PxMAP4K4*, *PxRaf*, *Pxp38*, *and PxERK* were upregulated in the midgut tissues of all resistant strains compared to the susceptible strain of *P. xylostella*. Increased phosphorylation of *MAP3K7*, Thousand and one amino acid (TAO) kinase, *MAP2K6*, *ERK*, and p38 was recorded in the resistant strain. In Bt resistant *P. xylostella* strain, upstream of these MAPKs, RAF is involved in ERK activation, TAO in JNK activation, and MAP3K7 in both p38 and JNK activation. *MAP4K4* activates all three key MAPKs of p38, JNK, and ERK (Guo et al., 2021).

Using CRISPR/Cas9, the contribution of two paralogous ABC transporters, *ABCC2* and *ABCC3*, and two aminopeptidases N, *APN1* and *APN3a* to Bt *Cry1Ac* toxicity in *P. xylostella* was evaluated. A knockout strain containing deleted *ABCC2* and *ABCC3* genes exhibited 4482-fold resistance to *Cry1A* toxin. Similarly, knockout strains with deleted *APN1* and *APN3a* genes exhibited 1425-fold resistance to *Cry1Ac* toxin, indicating their functional redundancy (Sun et al., 2022). Discovered that transcription factor *PxGATAd* activates membrane-bound *PxmALP* expression between the *Cry1Ac* susceptible and resistant strains by interacting with a non-canonical yet particular GATA-like cis-response element (CRE) present in the *PxmALP* promoter region to trigger the production of *PxmALP* directly. The ability of *PxGATAd* to regulate transcription was compromised by a six-nucleotide insertion mutation in this cis-acting area of the *PxmALP* promoter from the resistant strain. Additionally, *PxGATAd* silencing in susceptible larvae decreased *PxmALP* expression and sensitivity to *Cry1Ac* toxin. PxGATAd and *PxmALP* expression were both briefly restored when *PxMAP4K4* expression was suppressed in resistant larvae, demonstrating that *PxGATAd* is a positive, responsive factor that is involved in the activation of *PxmALP* promoter and negatively controlled by the MAPK signaling pathway (Guo L. et al., 2022).

Interesting to document is that *P. xylostella* has evolved a mechanism that makes it resistant to Bt toxins without impairing fitness. Fushi Tarazu Factor 1 (FTZ-F1), a MAPK-modulated transcription factor, downregulates Bt *Cry1Ac* toxin receptors while up-regulating non-receptor paralogs of MAPK cascades that regulate downstream transcription factors via the p38, Jun N-terminal kinase (JNK), or ERK pathways (Yang et al., 2013). Phosphorylated FTZ-F1 activates non-receptor genes through the motif “TAMAGTC,” whereas unphosphorylated FTZ-F1 initiates receptor genes via the binding site “YCAAGGYCR.” The activated MAPK cascade raises the amount of phosphorylation of FTZ-F1, conferring *P. xylostella* resistance to *Cry1Ac* toxin without impairing growth (Guo Z. et al., 2022). Host adaptation to the primary Bt virulence factors in *P. xylostella* was due to insertion of short interspersed nuclear element (SINE), SE2 in the promoter of *MAP4K4* gene that enhances the effect of transcription factor forkhead box O (FOXO) to induce MAPK pathway, which in turn potentiates host defense mechanism against the pathogen (Guo et al., 2023).

### GPCR/cAMP/PKA pathway

G protein-coupled receptors (GPCRs) are a large family of transmembrane receptors. It is a seven-transmembrane protein containing an extracellular domain for ligand binding and signal transduction into the cells regulating various cellular processes through an intracellular domain linked to a heterotrimeric G protein consisting of alpha (Gα), beta (Gβ), and gamma (Gγ) subunits. When GPCR interacts with its ligand, it induces cytosolic G-protein exchanges of guanosine diphosphate (GDP)-bound G protein for guanosine triphosphate (GTP). The G proteins are now separated into Gα and βγ-subunits (Gβγ). The Gα subunit triggers adenylate cyclase (Ac) to form the secondary messenger cAMP (cyclic adenosine monophosphate) from ATP, which then activates protein kinase A (PKA). PKA, in turn, induces a phosphorylation cascade that activates the cAMP-response element binding (CREB) transcription factor that finally regulates the expression of target genes (Hilger et al., 2018).

Caffeine induction of *CYP6A2* and *CYP6A8* in *D. melanogaster* was due to increased levels of cAMP due to the suppression of caffeine by cAMP-hydrolyzing cAMP phosphodiesterase (PDE) and downregulation of the AP-1 transcription factor Jun (Bhaskara et al., 2008). GPCR-related genes regulated the P450 gene expression in *Culex quinquefasciatus* (Diptera: Culicidae) for the first time where, suppressing GPCR with RNAi reduced permethrin resistance due to the decreased expression of related genes (*CYP6AA7*, *CYP9M10*, *CYP9J34*, and *CYP9J40*) in the high-resistance *C. quinquefasciatus* strain (Li T. et al., 2014).

The relative expression levels of the 94 GPCR genes in *M. domestica* among the near-isogenic imidacloprid resistance resistant strain (N-IRS), the susceptible strain (CSS) and another strain generated from field populations with imidacloprid resistance (IRS) were compared. It was found that compared to CSS strain, five GPCR genes were elevated in the N-IRS strain, and eight GPCR genes were upregulated in the IRS strains. Among the over-expressed GPCRs, LOC101899380 and LOC101895664 were heterologously expressed in *D. melanogaster*, and they were found upregulating *CYP6G1*, *CYP6A2*, *CYP6A8*, and *CYP12D1* genes. Moreover, the transgenic *D. melanogaster* created with the GPCR gene LOC101899380 affected the expression of all four P450 genes, but LOC101895664 only affected *CYP6G1* and *CYP6A2*, indicating that the latter is less involved in imidacloprid resistance via control of *CYP6A8* and *CYP12D1* expression (Ma et al., 2020).

The intermediary effectors engaged in insecticide resistance functions between GPCR020021 and the four target P450 genes, including 1 Gαs (Gαs006458), two AC (AC007240 and AC004739), and two PKAs (PKA000798 and PKA018257), were identified using a variety of functional genomics approaches. These include transgenic expression of GPCR020021 in *D. melanogaster* and heterologous expression of GPCR020021 and its downstream Gαs, AC, and PKA in Sf9 cells (Li and Liu, 2019) as well as *in vivo* RNAi knockdown of GPCR020021, Gαs006458, AC007240, AC004739, and the four target P450 genes in permethrin-susceptible (Li and Liu, 2018) and -resistant strains (Li et al., 2015; Li and Liu, 2017).

### Epitranscriptomic regulation of insecticide resistance

Epitranscriptome refers to different dynamic and reversible chemical modifications affecting RNA transcripts (coding and non-coding RNAs), which is the post-transcriptional regulation of gene expression. So, this structure and functions of dynamic RNA modifications during the developmental process and environmental stress and their effects on gene expression have emerged as a new branch of functional genomics known as “epi-transcriptomics.”

The dynamic and reversible RNA base modifications are catalyzed by enzymes like methyltransferases (writers) and removed by demethylases (erasers). Readers are the modification-specific binding proteins that interpret these modifications. It is similar to epigenetic DNA modification (Kumar et al., 2018). Many of the mRNA base modifications involve attachment of a methyl (CH3) group at a particular position either on the base [e.g., N6-methyladenosine (m6A), N1-methyladenosine (m1A), 5-methylcytidine (m5C), 3-methylcytidine (m3C), N7-methylguanosine (m7G), and 1-methylguanosine (m1G)], ribose sugar (e.g., 2-O-methyladenosine), or on both base and sugar [e.g., N6,2′-O-dimethyl adenosine (m6Am)] (Dominissini et al., 2016; Molinie et al., 2016).

m6A RNA is among eukaryotic mRNA’s most abundant chemical modifications. In *D. melanogaster*, the m6A pathway was involved in neuronal functions and sex determination (Lence et al., 2016). The transcriptome-wide profiling of m6A in the silkworm *Bombyx mori* has been used to identify its role in regulating gene expression, chromosome alignment and segregation, and nucleopolyhedrovirus (BmNPV) infection (Li B. et al., 2019).

*CYP4C64* has a crucial role in resistance to the neonicotinoid thiamethoxam and regulation of the gene by the m6A pathway in whitefly *B. tabaci* (Yang et al., 2021). *CYP4C64* was strongly over-expressed in the thiamethoxam-resistant strains compared with the susceptible strains. A polymorphism (T to A at position-206 bp) was observed in the 5′ UTR of *CYP4C64* at a greater frequency than 3′ UTR and CDS in the thiamethoxam-resistant strains compared with the susceptible strain. The T-206A transversion was predicted to have a sequence (CGACA) that resembles an N6 -methyladenine (m6A) sequence. The m6A modification on target RNAs, namely, methyltransferase-like 3 (*METTL3*) and 14 (*METTL14*), were confirmed in Western blot studies. *METTL3* was over-expressed in resistant strains compared with the susceptible strain. These results help us understand the epi-transcriptomic regulation of the xenobiotic response in insects and the role of m6A in developing insecticide resistance (Figure 5C).

### Future perspectives

For pest management or study, effective delivery mechanisms are required for introducing agomirs and antagomirs into insects. Feeding, injection, topical treatment, transgenic approaches, viral vectors, and nanoparticle-mediated delivery are all typical ways researchers introduce agomirs and antagomirs. Because insects are more receptive to external stimuli and may display more excellent feeding rates during this stage, the larval/nymphal stage is commonly selected for RNAi investigations linked to insecticide resistance (Jain et al., 2020). The incorporation of agomirs or antagomirs into the larval diet or by injection can result in efficient absorption and targeted gene silencing. For example, in the agomir and antagomir studies for insecticide resistance, fourth-instar nymphs of *N. lugens* (Li et al., 2022), third-instar larvae of P. *xylostella* (Zhu et al., 2020)*,* and 1-day post-emergence female mosquitoes (Sun et al., 2019) were often employed.

Recently, pest control using miRNA has been achieved through a process known as trans-kingdom RNA interference (TK-RNAi). It involves transfer of miRNAs through the diet, across kingdoms, to recipient organisms where they influence their biological activity. *Escherichia coli* has been engineered to express precursors for artificial miRNAs (amiRNAs) with insect targets for pest management by TK-RNAi via bacterial-mediated precursor miRNA expression. A reduction in oogenesis, significant mortality, and developmental abnormalities was seen in *H. armigera* larvae fed with E. coli-expressing a precursor for an RNA that targets *EcR* (Yogindran and Rajam, 2016). To control pests, another TK-RNAi method uses an insect or plant precursor backbone that expresses target amiRNAs or insect-specific miRNAs in transgenic plants. In transgenic tobacco plants, *Nicotiana tabacum* and *Nicotiana benthamiana*, expressing amiRNAs targeting *H. armigera* acetylcholinesterase, *AChE* 1 and *AChE* 2, respectively. Continuous feeding of first instar larvae with these plants led to increased mortality, developmental abnormalities, and delayed growth rates (Saini et al., 2018). Nymphs of the *B. tabaci* species exhibited aberrant egg hatching and poor development when they were raised on transgenic *N. tabacum* plants that expressed three separate amiRNAs (*amiRNASxl*, *amiRNAAChE*, and *amiRNAOrc*), which each target the *sex lethal protein* (*Sxl*), *AChE*, and *orcokinin* (*Orc*), respectively (Zubair et al., 2020). However, the stability of miRNA is a major concern to be considered. RNA chemical changes, such as ribose 20 hydroxyl group modification, unlocked or locked nucleic acids, and phosphorothioate backbone modification, have been researched to solve difficulties with miRNA stability. In comparison to a genetic modification strategy, these possible answers will be more practical with a spray technique.

## Conclusion

This review covered intricate cellular processes at the gene level in insecticide resistance responses as a survival mechanism in insects exposed to frequent insecticide applications in the agriculture and public health protection fronts. Recent outcomes on insecticide resistance research explored in high-throughput molecular methods demonstrate that resistance to insecticides has been linked to transcriptional activities of miRNAs and lncRNAs, a better understanding of these events that can be used in developing insecticide resistance management tactics. Substantial evidence has emerged on miRNAs regulating genes involved in detoxifying insecticides and modifying their target sites, the most common pesticide resistance strategies reported in insects.

By studying the miRNA profiles of insecticide-resistant insects, researchers can identify particular miRNAs that are upregulated or downregulated in response to insecticide exposure. Using these newly discovered miRNAs as potential therapeutic targets can reduce resistant populations and their expansions. Techniques for altering the expression or activity of these miRNAs can be developed to restore insecticide sensitivity or increase the efficacy of insecticide-based control strategies. Artificial mimics or inhibitors of specific miRNAs, for example, might modify their expression levels, potentially reversing or reducing pesticide resistance.

In the same way, lncRNAs have been linked to insecticide resistance affecting gene expression and biological processes by engaging with DNA, RNA, or proteins. lncRNAs are expressed differently in insecticide-resistant pests than in susceptible ones. These lncRNAs can regulate genes involved in insecticide detoxification, target site changes, or other resistance mechanisms. Understanding the roles and activities of these lncRNAs can give insights into the underlying molecular processes of insecticide resistance. It may lead to the development of resistance-fighting techniques against insects. Various technologies, such as RNA interference (RNAi) or gene editing techniques, can manipulate the expression or activity-specific lncRNAs. It may disrupt or regulate the regulatory networks involved in insecticide resistance by targeting resistance-associated lncRNAs, resulting in enhanced control strategies.

In conclusion, both miRNAs and lncRNAs can help manage insecticide resistance by providing intervention targets and a better knowledge of resistance mechanisms. However, it is essential to emphasize that RNA-based pest control is still in its early stages, and more study is needed to properly investigate and harness the potential of miRNAs and lncRNAs in countering insecticide resistance.

m6A is a well-known and thoroughly researched RNA modification cellular process in organisms. The m6A modification modulates RNA metabolism, such as mRNA stability, splicing, and translation. A recent study has shown that m6A mutations have a role in insecticide resistance in some insect species. Changes in the m6A landscape have been detected in resistant insect populations, indicating a potential function in modifying resistance-related gene expression.

Researchers may gain insight into the regulatory processes driving resistance development by examining the epi-transcriptomic alterations associated with insecticide resistance. Understanding how particular RNA changes impact the expression and function of resistance genes might give helpful knowledge for creating resistance-fighting tactics. Furthermore, altering RNA changes might be a focused way to manage insecticide resistance. It may modulate the expression of resistance-related genes and restore insecticide sensitivity by regulating the enzymes responsible for adding or deleting particular RNA modifications.

However, it is essential to highlight that the epitranscriptomics and insecticide resistance area is still in its early phases. More study is needed to understand the exact epi-transcriptomic changes associated with resistance and their functional implications. Furthermore, finding tools and procedures for precise manipulation of RNA changes in insects is a problem that must be overcome. Continued study in this area will help us understand the epitranscriptomic control of pesticide resistance and may lead to novel techniques for managing resistant pest populations.

Researchers can uncover crucial molecular targets and propose creative techniques to manage resistance by unraveling the regulatory processes linked with insecticide resistance. Combining insecticides with synergists that inhibit detoxifying enzymes, modifying essential regulatory proteins or pathways via RNA interference or gene editing technologies, and developing novel insecticides that target specific resistance mechanisms are all examples of such methods. It is critical to note that resistance regulatory mechanisms vary and develop between pest species and populations. Continuous research and observations are essential to stay tuned in and gather more profound information on the insecticide resistance phenomena and build sustainable management approaches.

## Figures and Tables

**Table:** 

**ABC**	ATP-Binding cassettes
**ABCG**	ATP-Binding cassettes group
***ACC***	Acetyl-CoA carboxylase
**AChE**	Acetylcholine esterase
**AhR**	Aryl hydrocarbon Receptor
**AKH**	Adipokinetic hormone
**ARE**	Antioxidant Response Element
**ARNT**	Aryl Hydrocarbon Receptor Nuclear Translocator
***BPH***	Brown planthopper
**bZIP**	Basic leucine zipper
**CarE**	Carboxyl esterase
**CCE**	Carboxyl choline esterase
**CDS**	Coding sequence
**CncC/Keap1**	Cap'n'collar isoform C/Kelch-like ECH associated protein 1
**CNV**	Copy Number Variation
**CYP**	Cytochrome P450
**CyR**	Cyflumetofen-resistant strain of *T. cinnabarinus*
**DBD**	DNA binding domain
**DDT**	Dichloro-diphenyl-trichloroethane
**DHR96**	*Drosophila* Hormone receptor-like in 96 (HR96)
**EcR**	Ecdysone receptor
**ERK**	Extracellular Signal-Regulated Kinase
**EST**	Esterase
**FTZ**	Fushi Tarazu
**GPCR**	G protein-coupled receptor
**GST**	Glutathione S Transferases
**HNF**	Hepatocyte nuclear factor
**HR96**	Hormone receptor-like in 96
**IR**	Insecticide resistance
**LBD**	Ligand binding domain
**Maf**	Muscle aponeurosis fibromatosis
**MAPK**	Mitogen-activated phosphate kinase pathway
***METTL***	Methyltransferase-like
**MFO**	Mixed function oxidase
**miR**	Mature microRNA
**NGS**	Next-generation sequencing
**NR**	Nuclear receptor
**Nrf2**	NF-E2-Related Factor 2
***PxEcR-B***	*Plutella xylostella* Ecdysone receptor isoform B
***PxJHE***	*Plutella xylostella* juvenile hormone esterase
***PxRyR***	*Plutella xylostella* ryanodine receptor
**RAS**	Renin-angiotensin system
**RAF**	Rapidly Accelerated Fibrosarcoma
**RISC**	RNA induced silencing complex
**RTK**	Receptor tyrosine kinases
**RNA**	Ribo Nucleic acid
**RNAi**	RNA interference
**ROS**	Reactive oxygen species
**RXR**	Retinoid-X-receptor
**RyR**	Ryanodine receptor
***SBPH***	Small backed planthopper
**SXR/PXR**	Steroid and xenobiotic receptor/Pregnane X Receptor
**TAO**	Thousand and one amino acid
**USP**	Ultraspiracle protein
**UTR**	Untranslated region
**VGSC**	Voltage-gated sodium channel
**XRE**	Xenobiotic Response Element
