# Supplementary material for: Enthralling genetic regulatory mechanisms meddling insecticide resistance development in insects: role of transcriptional and post-transcriptional events
Source: Front Mol Biosci. 2023 Sep 6;10:1257859. doi: 10.3389/fmolb.2023.1257859 (PMC10511911; doi:10.3389/fmolb.2023.1257859)
Supplement: Supplementary file 1 [file Table1.DOCX]

**Table S1**  List of insect microRNAs involved in detoxification of and resistance to different insecticides

| **MicroRNA** | **MicroRNA Regulation** | **Target gene** | **Target gene**  **regulation** | **Species** | **Function** | **Confirmatory study** | **References** |
| --- | --- | --- | --- | --- | --- | --- | --- |
| miR-932 | Up | *CPR5* | Down | *Culex pipiens pallens* | Deltamethrin resistance | Molecular- Expression studies | (Liu et al., 2016) |
| miR-278-3p  miR-2  miR-285  miR-71  miR-13 | Down  Down  Down  Down  Up | *CYP6AG11*  *CYP9J35*  *CYP6N23 CYP6Cp1*  *CYP325BG3*  *CYP9J35* | Up  Up  Up  Up  Down | *C. pipiens pallens* | Deltamethrin resistance | Expression analysis | (Fahmy et al., 2020) |
| miR-4448 | Down | *CYP4H31* | Up | *C. pipiens pallens* | Deltamethrin resistance | Bioinformatics and molecular | (Li et al., 2021a) |
| miR-13664 | Down | *CYP314A1* | Up | *C. pipiens pallens* | Deltamethrin resistance | Bioinformatics, mimics, and inhibitor study | (Sun et al., 2019) |
| let-7 | Down | *CYP6CY3* | Up | *Myzus persicae* | Detoxification | Bioinformatics and expression analysis | (Peng et al., 2016) |
| miR-100 | Down | *CYP6CY3* | Up | *M.persicae* | Detoxification | Bioinformatics and expression analysis | (Peng et al., 2016) |
| miR-276 | Down | *ACC* | Up | *Aphis gossypii* | Spirotetramat resistance | Expression studies | (Wei et al., 2016) |
| miR-3016 | Down | *ACC* | Up | *A. gossypii* | Spirotetramat resistance | Expression studies | (Wei et al., 2016) |
| miR-4133-3p | Up | *CYP4CJ1* | Down | *A. gossypii* | Detoxification | Bioinformatics and expression analysis | (Ma et al., 2019) |
| miR-316 | Down | *CYP4CJ6* | Up | *Sitobion miscanthi* | Imidacloprid resistance | Expression analysis | (Zhang et al., 2022a) |
| miR-278 | Up | *nAChRα1A* | Down | *S. miscanthi* | Imidacloprid resistance | Expression analysis | (Zhang et al., 2022a) |
| miR-263b | Up | *nAChRβ1* | down | *S. miscanthi* | Imidacloprid resistance | Molecular- transcriptome and DGE | (Zhang et al., 2022a) |
| miR-8533-3p  miR-8534-5p  miR-375-5p | Down | *LCP-30*  *CYP6B6*  *CYP4G15* | Up | *P. xylostella* | Chlorantraniliprole resistance | Bioinformatics and expression analysis | (Zhu et al., 2017) |
| miR-2b-3p, miR14b-5p  let-7-5p | Down | *CYP9F2 CYP307a1* | Up | *P. xylostella* | Chlorantraniliprole resistance | Bioinformatics and expression analysis | (Etebari et al., 2018) |
| miR-189942 | Up | *EcR-B* | Down | *P. xylostella* | Fufenozide resistance | Bioinformatics and expression analysis | (Li et al., 2020) |
| miR-998-3p | Down | *ABCC2* | Up | *P. xylostella* | Bt*Cry1Ac* toxin | Bioinformatics and expression analysis | (Zhu et al., 2020) |
| miR-8525-5p | Down | *GSTu1*  *lnc-GSTu1-AS* | Up | *P. xylostella* | chlorantraniliprole resistance | Bioinformatics and molecular- RNAi | (Zhu et al., 2021) |
| novel-miR-310 | Up | *PxABCG20* | Down | *P. xylostella* | Bt*Cry1Ac* toxin | Bioinformatics and molecular | (Yang et al., 2022a) |
| miRNA novel_268 | Down | *NlABCG3* | Up | *Nilaparvata*  *lugens* | Nitenpyram and Clothianidin | Molecular-RNAi | (Li et al., 2022) |
| Novel_85  Novel_191 | Down | *CYP6ER1*  *CarE1* | Up | *N. lugens* | Nitenpyram | Bioinformatics and molecular | (Mao et al., 2022) |
| miR-190-5p | Down | *CYP6K2* | Up | *Spodoptera frugiperda* | Chlorantraniliprole resistance | Molecular analysis- RNAi | (Zhang et al., 2022b) |
| miR-1-3p | Down | *TCGSTM4* | Up | *T. cinnabarinus* | cyflumetofen resistance | Molecular analysis- RNAi | (Zhang et al., 2018) |
| miR-133-5p  lincRNA_Tc13743.2 | Down  Up | *TcGSTm02* | Up | *T. cinnabarinus* | cyflumetofen resistance | Expression and co-expression analysis | (Feng et al., 2020) |
| miR-310-3p  miR-311-3p  miR-312-3p  miR-313-3  miR-92a-3p | Down  Down  Down  Down  Down | *CYP6G1*  *CYP6G2*  *CYP6G1*  *CYP6G2*  *CYP6G1*  *CYP6A8*  *CYP6G1*  *CYP6G2*  *CYP4G1*  *CYP6A8*  *CYP6G2*  *CYP4G1* | Up  Up  Up  Up  Up | *Drosophila melanogaster* | DDT | Expression and co-expression analysis | (Seong et al., 2019) |
| novel-miR-4 | Up | *CYP4C1* | Down | *Lymantria dispar* | Cyantraniliprole | Integrated MiRNA-m RNA expression analysis | (Zhang et al., 2023) |
