# Supplementary material for: Enthralling genetic regulatory mechanisms meddling insecticide resistance development in insects: role of transcriptional and post-transcriptional events
Source: Front Mol Biosci. 2023 Sep 6;10:1257859. doi: 10.3389/fmolb.2023.1257859 (PMC10511911; doi:10.3389/fmolb.2023.1257859)
Supplement: Supplementary file 2 [file Table2.DOCX]

**Table S2** Transcription factors and their corresponding regulatory pathways involved in insecticide detoxification

| **Insect** | **Detoxification gene** | **Transcription factors** | **Insecticide** | **IRAC target** | **References** |
| --- | --- | --- | --- | --- | --- |
| *Spodoptera exigua* | *GSTo2*, *GSTe6* and GSTd3 | CncC/Maf | Chlorpyrifos  Cypermethrin | Acetylcholinesterase (AChE) inhibitors (Group 1B)  Sodium channel modulators (Group 3A) | Hu *et al*., 2019 |
| *S. exigua* | *GSTo2* and *GSTe6* | AhR/ARNT | Chlorpyrifos  Cypermethrin | Acetylcholinesterase (AChE) inhibitors (Group 1B)  Sodium channel modulators (Group 3A) | Hu *et al*., 2019 |
| *S. exigua* | *CYP321A16* and *CYP332A1* | CncC and Maf | Chlorpyrifos | Acetylcholinesterase (AChE) inhibitors (Group 1B) | Bo *et al*., 2020 |
| *S. exigua* | *CYP321A8* | CncC/Maf, orphan nuclear receptor- Knirps | Chlorpyrifos  Cypermethrin  Deltamethrin | Acetylcholinesterase (AChE) inhibitors (Group 1B)  Sodium channel modulators (Group 3A) | Hu *et al*., 2021 |
| *Spodoptera litura* | *CYP367A1*, *CYP367B1*, *CYP341B21*, *CYP340L2*, *SlituCXE1*, and *SlituABCH-1* | CncC–Maf | Indoxacarb | Voltage-dependent sodium channel blockers (Group 22) | Shi *et al*., 2021 |
| *S. litura* | *CYP6AB12* | CncC and Maf | λ-Cyhalothrin | Sodium channel modulators (Group 3A) | Lu *et al*., 2020 |
| *Plutella xylostella* | *CYP6BG1* | FTZ-F1 | Chlorantraniliprole | Ryanodine receptor modulators (Group 28) | Li *et al*., 2019b |
| *Helicoverpa armigera* | *CYP321A1* | CncC | Flavone | - | Zhang *et al*., 2023c |
| *Dendroctonus armandi* | *CYP4BQ1* | ROS/CncC | Pinene | - | Liu *et al*., 2022 |
| *Tribolium castaneum* | *CYP4Q4*, *CYP4G7*, *CYP4BR3*, and *CYP345A1* | HR96 | Imidacloprid | Nicotinic acetylcholine receptor (nAChR) competitive modulators (Group 4) | Kim *et al*., 2021 |
| *Leptinotarsa decemlineata* | *CYP6BJ1*, *CY9Z26*, *GST-3*, *ABCH278B*, *ABCH278C*, and *ABCG1041A* | CncC | Imidacloprid | Nicotinic acetylcholine receptor (nAChR) competitive modulators (Group 4) | Gaddelapati *et al*., 2018 |
| *L. decemlineata* | CYP6BJa/b 35, CYP6BJ1v1, CYP9Z25, and CYP9Z29 | Cncc/maf | Potato plant allelochemicals  Imidacloprid | Detoxification  Nicotinic acetylcholine receptor (nAChR) competitive modulators (Group 4) | Kalsi and Palli, 2017 |
| *Aphis gossypii* | *CYP6DA2* | Cncc | Gossypol | - | Peng *et al*., 2016 |
| *A. gossypii* | *CYP6DA2* | AhR and ARNT | Gossypol | - | Peng *et al*., 2017 |
| *Bemisia tabaci* | *CYP6CM1* | MAPK-CREB | Imidacloprid | Nicotinic acetylcholine receptor (nAChR) competitive modulators (Group 4) | Yang *et al*., 2020 |
| *Nilaparvata lugens* | *CYP301A1*, *GSTt1* and *CarE7* | AhR and ARNT | Imidacloprid  Etofenprox  Isoprocarb | Nicotinic acetylcholine receptor (nAChR) competitive modulators (Group 4)  Sodium channel modulators (Group 3A)  Acetylcholinesterase (AChE) inhibitors (Group 1B) | Wang *et al*., 2021 |
| *N. lugens* | *UGT-2B10*, *CYP4CE1*, *CarE*and *Esterase E4-1* | NR2E nuclear receptor HR83 | Chlorpyrifos | Acetylcholinesterase (AChE) inhibitors (Group 1B) | Lu *et al*., 2021 |
| *Anopheles gambiae* | *CYP6M2* and *GSTD1* | Maf-S | Permethrin  Deltamethrin  DDT | Sodium channel modulators (Group 3A)  Sodium channel modulators  (Group 3B) | Ingham *et al*., 2017 |
| *Bactrocera dorsalis* | *GSTz2*, and *CYP473A3* | *MafB* | Abamectin | Glutamate-gated chloride channel (GluCl) allosteric modulators (Group 6) | Tang *et al*., 2019 |
| *Tetranychus cinnabarinus* | *CYP389B1*, *CYP391A1* and *CYP392A28* | CncC and Maf | Fenpropathrin | Sodium channel modulators (Group 3A) | Shi *et al*., 2017 |
| *Locusta migratoria* | *GSTd7* | AhR | Chlorpyrifos | Acetylcholinesterase (AChE) inhibitors (Group 1B) | Zhang *et al*., 2019 |
